# Valorization of Invasive Plants from Macaronesia as Filler Materials in the Production of Natural Fiber Composites by Rotational Molding

**DOI:** 10.3390/polym13132220

**Published:** 2021-07-05

**Authors:** Zaida Ortega, Francisco Romero, Rubén Paz, Luis Suárez, Antonio Nizardo Benítez, María Dolores Marrero

**Affiliations:** 1 Departamento de Ingeniería de Procesos, Universidad de Las Palmas de Gran Canaria, 35017 Las Palmas, Spain; antonionizardo.benitez@ulpgc.es; 2 Departamento de Ingeniería Mecánica, Universidad de Las Palmas de Gran Canaria, 35017 Las Palmas, Spain; francisco.romero@ulpgc.es (F.R.); ruben.paz@ulpgc.es (R.P.); luis.suarez@ulpgc.es (L.S.); mariadolores.marrero@ulpgc.es (M.D.M.)

**Keywords:** rotational molding, polyethylene, natural fibers, composites, mechanical properties, particle size distribution, sieving, invasive plant species

## Abstract

This paper compares the mechanical properties of different natural fiber composites produced by rotational molding as a way of waste valorization from campaigns to control invasive plant species in Macaronesia. Rotomolded parts produced with polymeric matrices (polyethylene) and filled with up to 20% by weight of cellulosic fibers obtained from *Arundo donax* L., *Pennisetum setaceum*, and *Ricinus communis* plants were characterized in terms of tensile, flexural, and impact strength. It was found that the sieving of natural fibers allowed for their introduction in higher loadings, from 10 (for un-sieved material) to 20%; fiber size greatly affected the mechanical properties of the final parts, although some combinations were proven not to reduce the mechanical properties of the neat resin. This study is a first approach to the valorization of residues obtained from periodic campaigns of the control of invasive species performed by public authorities, usually at the local level. It is important to highlight that the main objective of this research did not focus on economically profitable activity; instead, it was focused on the reduction of wastes to be disposed from ecosystem maintenance actions and the investment of potential income into preservation policies.

## 1. Introduction

Invasive species are a real threat to the preservation of ecosystems, with their presence being especially dangerous in areas with a high biodiversity such as the Macaronesia archipelagos: Madeira, Azores, and the Canary Islands. Some plant species such as the giant reed (*Arundo donax* L.), fountain grass (*Pennisetum setaceum*), and castor oil plant (*Ricinus communis*) have spread without any control in these three archipelagos, ultimately affecting natural ecosystems and endangering some of the endemic species found in this region. The Macaronesia region, despite representing only 0.2% of the of the European Union’s territory, hosts over a quarter of the plant species of community interest listed in Annex II of the Habitats Directive (92/43/EEC) [1]. These plants, among others, are included in the Spanish catalogue of invasive species produced by the Ministry of Agriculture and Environment [2], as well as in the list of invasive plants of the Canary Islands Government [3]; some of them, such as the giant reed, are also included in the top 100 most dangerous species of the International Union for Conservation of Nature (IUCN) [4]. These plants produce changes in the hydrological regime, compete with endemic species in resources, vary soil conditions (salinity and pH), and contribute to fire propagation due to the large amount of biomass they produce.

Several campaigns are undertaken every year to control the spread of these specimens on the Canary Islands, but these do not appear to be fully effective in controlling and eradicating these invasive species, mainly due to a lack of regularity. As a way of increasing the effectiveness of these control strategies, the Inv2Mac project (funded by Interreg MAC 2014–2020 program, grant number MAC2/4.6d/229) has proposed the use of the plant waste from these campaigns to obtain natural fibers for their use in composite production. The main aim of this project consists of achieving a better environmental state of preservation of natural ecosystems by studying the distribution of different invasive plant species in Macaronesia; performing their biological, chemical, and physical characterization; and trying to valorize them to obtain composite materials—with the final objective of supporting periodic actions of ecosystem cleaning.

**Figure 1:**
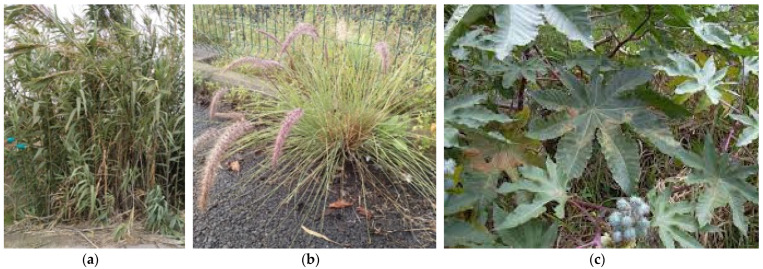
Species used in the research: (**a**) *Arundo donax* L., (**b**) *Pennisetum setaceum*, and (**c**) *Ricinus communis*.

In particular, this work deals with the use of cellulosic fibers obtained from *Arundo donax* L. (AD), *Pennisetum setaceum* (PS), and *Ricinus communis* (RC) (Figure 1) for the production of polymer matrix composites by rotational molding. in summary of the bio-distribution study, it can be concluded that the giant reed and castor oil plant can be found in the three archipelagos, though the latter is only found in anthropized spaces. Fountain grass can only be considered dangerous in the Canary Islands, although some first populations have been found in Madeira. This study is a first approach to the valorization of residues obtained from periodic campaigns of the control of invasive species, performed by public authorities, usually at the local level. It is important to highlight that the main objective of this research did not focus on economically profitable activity; instead, it was focused on the reduction of wastes to be disposed from ecosystem maintenance actions and the investment of potential income into preservation policies.

It is well known that natural fibers have experienced a great rise in interest in the last few years due to their renewable character, low price, good specific properties, and continuously growing market [5]. The increased demand for sisal, ramie, and curaua fibers due to their good mechanical properties has contributed to the market growth. Natural fibers are used in different sectors, among which the composites industry is the most relevant to this research.

The giant reed has been used for several applications, such as raw material in biorefineries [6], filters for heavy metal separation [7], structural elements for bio-construction [8] or isolating panels [9], and as a reinforcement [10,11,12,13]. García-Ortuño et al. used the chipped giant reed in a urea-formaldehyde matrix to obtain compression molded panels [14] with low thermal conductivity and mechanical properties suitable for furniture building. Fiore et al. [12] processed the culm of the giant reed (the stem) for use as fillers in PLA and epoxy resin matrices [10,15]. Up to 15% of *Arundo* was used for the epoxy composites (by infusion) and up to 20% of *Arundo* was used for PLA resin (by compression molding). The elastic modulus was found to be improved due to the introduction of the natural fibers, while strength was reduced for epoxy composites. Plasma treatment seemed to increase the flexural properties of the epoxy composite (with 5% fiber), according to Scalici [11]. When used as reinforcement in a polyurethane composite, NaOH treatment also improved the mechanical properties of the fibers, achieving up to 40% fiber loading.

*Ricinus* is commercially grown for oil obtainment with a wide variety of applications, such as lubricants, hydraulic fluids, plastics, and pharmaceutic products. *Ricinus* oil is obtained from the seeds of the plant, with a yearly production of over a million tons [16]. Unfortunately, *Ricinus* seeds are only a small part of the plant, with the leaves and stems being by-products that could be used in several applications, such as bio-absorbents [17]. The liberian fibers of *Ricinus* comprise around 10–20% of the entire stem [18]. Regarding the use of *Ricinus* in composites, Heitzmann obtained 30% composites (in volume) using PA11 [19], also obtained from the same plant (Rilsan from Naturelle) via the compression molding process. Flexural properties are similar to those obtained for the same matrix and glass fiber, with the comparison being favorable to *Ricinus* fiber when considering specific properties (while considering the composite weight). Vinayaka obtained a composite with 50% of *Ricinus* fibers in a polypropylene matrix via the compression molding of hybrid mats (made of PP and *Ricinus* fibers) [20].

Finally, no studies on the use of *Pennisetum setaceum* for composite obtainment have been found; only some experiences in transforming this plant into paper pulp have been found [21].

On the other hand, rotational molding is a widely used process to obtain hollow parts, with a good superficial quality, good thickness distribution, and good mechanical properties. The main disadvantages of this process are its long cycle time, low energy efficiency, and the low availability of materials suitable to be processed by this technology [22]. The injection or compression molding of natural fiber-reinforced composites has been widely studied, but just a few studies on using these types of fibers for rotational molding have been found due to the high sensibility of this process to the introduction of foreign materials, reducing the mechanical properties of the obtained part—mainly the impact strength—in a significant way [23]. Together with the use of bio-based, biodegradable, or recycled polymers [24], the incorporation of natural fibers into polymer matrices is seen as a way to improve the environmental behavior of plastic parts, as part of the polymer is substituted by a material coming from a renewable resource.

Regarding the mechanical properties of natural fiber composites, the use of a composite containing 10% in weight of flax fiber [25] does not modify the tensile or impact strength of samples. Preparing composites with cabuya and sisal does not improve the tensile properties of polyethylene, and a significant reduction in impact properties occurs [26]. Furthermore, the use of foreign materials, such as fibers, in a low shear process results in low mixing, which does not improve the mechanical properties of the resin because fibers separate from the powder and lay on the inner surface of the manufactured part [27]. The presence of voids and defects in a composite prevents good contact between the fiber and matrix, thus reducing nucleating effect (and thus leading to lower crystallinity values), which also explains the worse mechanical properties that are usually found in rotomolded composites, especially for those with high fiber contents [28]. In this context, we present a novel research work that studied the use of fibers from invasive plant species in rotational molding, as well as the influence of their size on the mechanical properties of the composites, by incorporating up to 20% of these fibers into a polyethylene (PE) matrix, which is the most common material in rotomolding [29].

## 2. Materials and Methods

General purpose PE from Matrix (Revolve N-461, Northampton, UK), in powder form, was used in all the experiments. The polymer was manually mixed with the appropriate amount of fiber to obtain a 20% of loading in the final composite.

Fibers from the giant reed (*Arundo donax* L., AD) were obtained from plants with an average stem diameter of 2 cm. The stems were then submitted to a series of crushing, knurling, and V-shaped rollers in order to remove fine particles and separate the stem into strips. A similar process, based on a tooth roller, was used to break the external layer of fountain grass stems (*Pennisetum setaceum*, PS). The vegetal material thus obtained was then chopped to around 2 mm length in a lab-made cutting machine prior to milling in a Retsch ZM 200 mill (Haan, Germany). Finally, for the castor oil plant (*Ricinus communis*, RC), the stems were directly chopped and milled in the mill. The resulting materials were then placed into sieving equipment AS 200 Control, also from Retsch (Haan, Germany), with sieves of 75, 125, and 250 μm separating four different fractions: A (>250 μm), B (125–250 μm), C (75–125 μm), and D (<75 μm). Sieving took place for 5 min, with an oscillation amplitude of 1.00 mm/g at intervals of 30 s.

Once the fibers were milled, they were soaked in an NaOH 1N solution for 1 h in order to improve their thermal stability. They were washed with water until reaching neutral pH and then dried in an oven at 105 °C for 12 h; once dried, they were sieved to the above-mentioned fractions (sizes A–D). These fibers (and their composites) are referred to in this paper with a “t” (i.e., treated *Arundo* is shortened to ADt).

Table 1 summarizes the composites developed in this work and the corresponding codes. Note that for the sake of simplicity, the treated fibers were not included, but the code would be the same but with an additional “t” after the fiber type. For example, R.PE.PSt.5 corresponds to rotomolded PE with 5 wt% *Pennisetum setaceum*-treated fiber (non-sieved).

**Figure 2:**
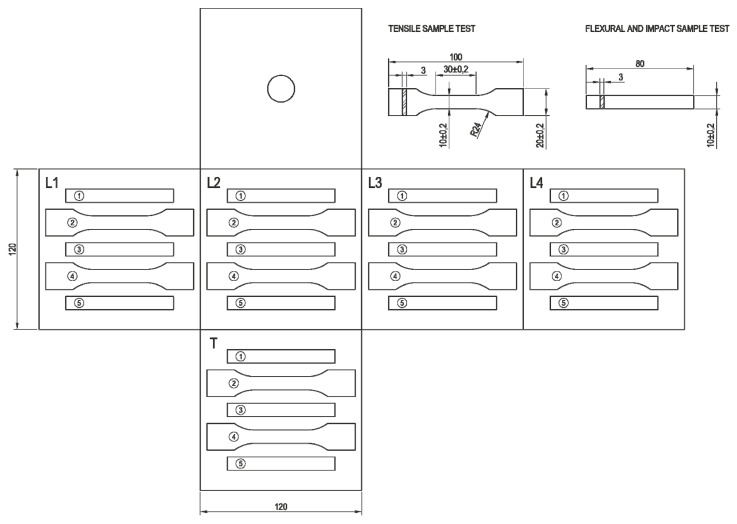
Distribution of test samples in the rotomolded cube faces.

Materials were introduced in an aluminum cube-shaped mold (120 mm side) that was placed in a lab-made rotomolding machine (clamshell type with air convection heating/cooling system), introducing enough material to get a part with 3–4 mm thickness. The mold was monitored (internal air temperature) with a thermocouple introduced via the venting hole placed in the middle of one side of the cube. The fan in the oven was set to 1200 L/min (normal liters) and a temperature of 320 °C, and the heating stage was ended when the temperature inside the mold reached 180 °C. The rotation speed was 2.30 rpm in the primary axis and 9.14 in the secondary axis (speed ratio of 1/4 primary axis/secondary axis, respectively). Once the cubes were extracted from the mold, they were measured in their three main dimensions to determine shrinkage using a Mitutoyo caliper. Then, the cubes were cut with a belt saw to obtain six faces from which test samples were machined in a machining center following the distribution shown in Figure 2. The geometry of the test samples was in accordance with UNE-EN ISO 3167 standards for tensile tests, UNE-EN ISO 178 standards for flexural tests, and UNE-EN ISO 180 standards for Izod-impact tests.

These samples were tested to determine their tensile behavior, i.e., elastic modulus, elastic limit, and maximum tensile strength, according to UNE-EN 527–2:2012, at a rate of 10 mm/min. Flexural properties were measured according to UNE-EN ISO 178:2011 at the same rate, with a distance between cantilevers of 64 mm; ultimately, the elastic modulus, elastic limit, and maximum flexural strength were obtained. Impact resistance values (impact energy absorbed by the part, referred to the initial cross section of the bar) were determined following UNE-EN ISO 180:2001/A2:2013 (Izod) using unnotched samples (ISO 180/U), a pendulum of 5.5 J, and an impact rate of 3.5 m/s. Tensile and flexural tests were performed in a testing machine from Dongguan Liyi Test Equipment Co. Ltd. (Dongguan, China, model LY-1065), while impact properties were determined in an Izod and Charpy impact tester model LY-XJJD 50 from Dongguan Liyi Test Equipment Co. Ltd. (China).

Test bars were metalized over the breakage section (using an Au/Pd target in a sputtering SC 760 apparatus from Quorum Technologies, Lewes, England) for 120 s and 18 mA under argon atmosphere. Samples were then introduced to a TM3030 tabletop scanning electron microscope from Hitachi (Tokyo, Japan) working at 15 kV.

## 3. Results

The segregation and agglomeration of fillers and reinforcing materials have been recurrent problems in the production of composites by rotomolding [30]. After preparing and characterizing different formulations of rotational molding composites with fiber contents from 5 to 10% (wt), the possibility of increasing the amount of filler material to 20% (wt) by grinding and sieving the natural fibers was assessed.

### 3.1. Composites with Untreated Fibers

#### 3.1.1. Composites Obtained with *Arundo donax* L.

**Figure 3:**
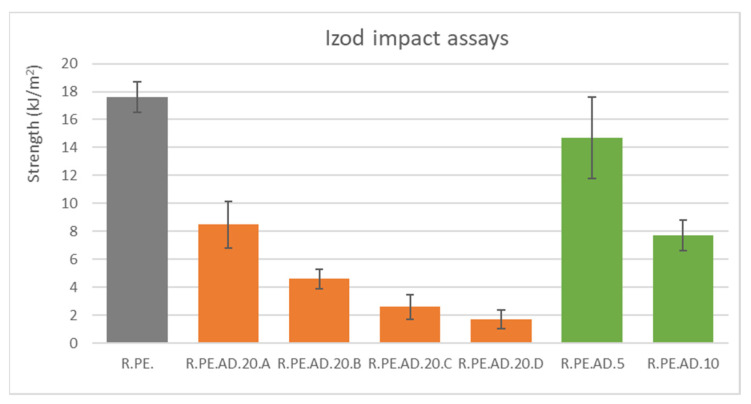
Izod impact assays results for *Arundo* composites.

Figure 3 shows results from the impact assays of composites with the giant reed. As expected, impact strength was greatly affected by the incorporation of the vegetal fillers into the PE matrix. It was also observed that the bigger the filler, the lower the reduction in this property. Additionally, composites with 20% fiber and sizes larger than 250 μm showed similar results to those obtained for 10% composites obtained without sieving the fibers.

The density of natural fiber composites is one of the properties that is often measured in order to ascertain specific properties by dividing the value of the measured property by the density. The introduction of *Arundo* fibers allowed for the obtainment of composites with densities reduced by almost 40% for the 20% composites (all data are compiled in Table S1) compared to the samples entirely made of PE. If the density of the composite was considered, the loss in impact strength was found to be reduced; we were able to produce a composite with 60.4% of the specific impact strength of neat PE (vs. the 48.3% found without considering density). In any case, the composites with a smaller fiber size (smaller than 75 μm) only showed a tenth of the impact strength of the PE (and almost 15% of the specific impact strength).

**Figure 4:**
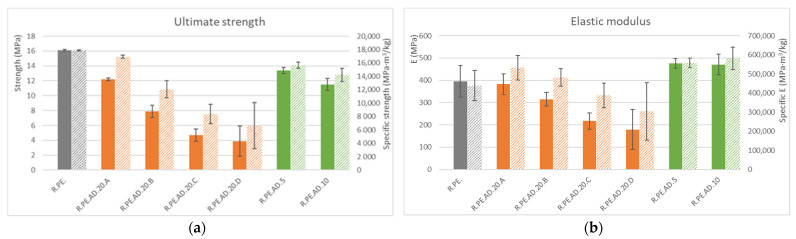
(**a**): tensile ultimate strength (solid color) and specific ultimate strength (lined) for *Arundo* composites; (**b**): tensile elastic modulus (solid color) and specific elastic modulus (lined).

The difference in using absolute or specific properties is clearer in Figure 4, where average values for ultimate strength (at breaking in the tensile test) are shown. Strength was reduced for all composites containing *Arundo*, although the material with the bigger particle size showed a specific strength similar to that of neat PE.

Similar observations could be made for elastic modulus, although the reduction for this property was smaller than for other properties, and some reinforcement was even seen for low amounts of *Arundo* (5 and 10%), which improved elastic modulus by nearly 20%. It is interesting to highlight that, again, 20% composites with large amounts of particles allowed us to obtain a composite with the same elastic modulus of neat PE, along with a 20% higher specific modulus and a similar value to those of composites with low fiber contents. This demonstrated that fiber sieving before processing by rotational molding allows for the obtainment of better specific mechanical properties than when using the vegetal material as obtained; we were ultimately able to increase the amount of this material by up to 20%, which is not often seen in this technology.

**Figure 5:**
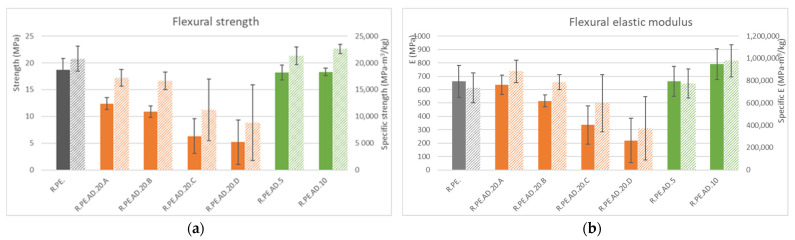
(**a**): flexural ultimate strength (in solid color) and specific ultimate strength (lined) for *Arundo* composites; (**b**): flexural elastic modulus (in solid color) and specific elastic modulus (lined).

**Figure 6:**
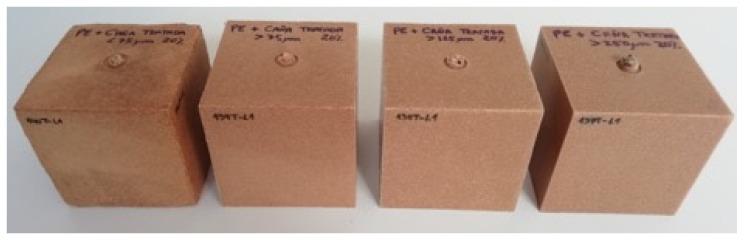
Rotomolded cubes with 20% weight of *Arundo donax* L. at different fiber sizes: <75 μm (size D), 75–125 μm (size C), 125–250 μm (size B), and >250 μm (size A) (from left to right, respectively).

Flexural tests (summarized in Figure 5) showed a similar trend to that observed for tensile tests: the shorter the fibers, the lowermechanical properties obtained and the larger the observed deviations. These increased deviations could be attributed to the tendency of shorter fibers to agglomerate, thus hindering their distribution inside the polymer matrix and reducing the homogeneity of the obtained part, as can be observed in Figure 6.

#### 3.1.2. Composites Obtained with *Pennisetum Setaceum*

**Figure 7:**
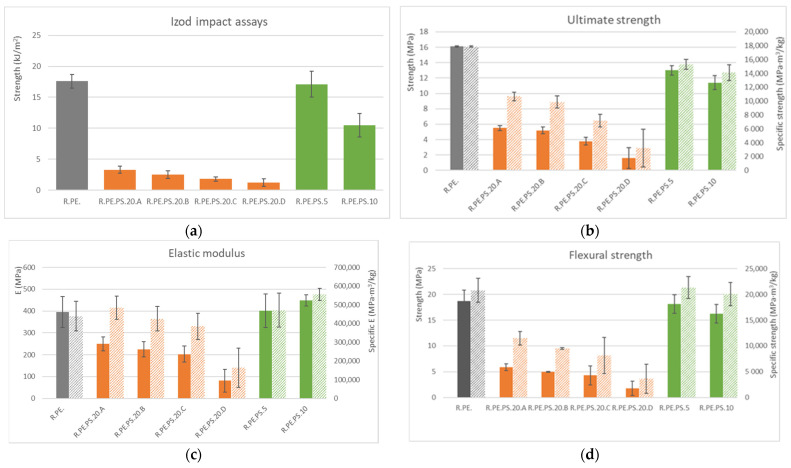

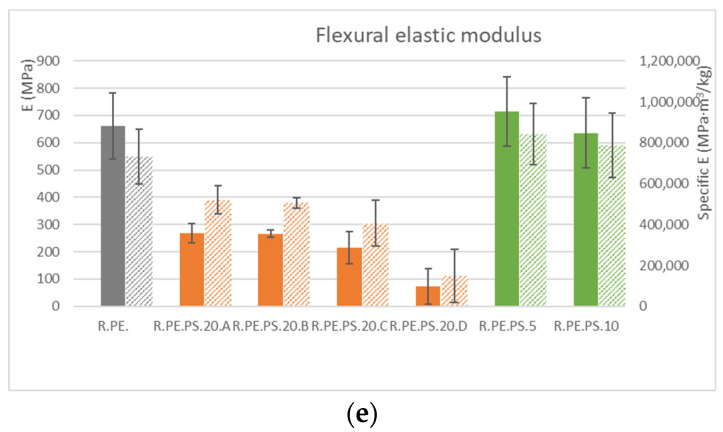
Summary of mechanical properties obtained for rotomolded *Pennisetum setaceum* composites (solid bars show absolute properties, and lined bars show specific properties): (**a**) Izod impact assays, (**b**) tensile ultimate strength, (**c**) tensile elastic modulus, (**d**) flexural maximum strength, (**e**) flexural elastic modulus.

Figure 7 shows results obtained from the mechanical tests of composites with *Pennisetum*; poor mechanical properties were obtained when using this plant as filler for loadings over 5% wt, even when analyzing specific properties, except for tensile elastic modulus. A similar behavior to *Arundo* composites was observed for natural loading size, i.e., smaller particles sizes resulted in worse mechanical properties.

**Figure 8:**
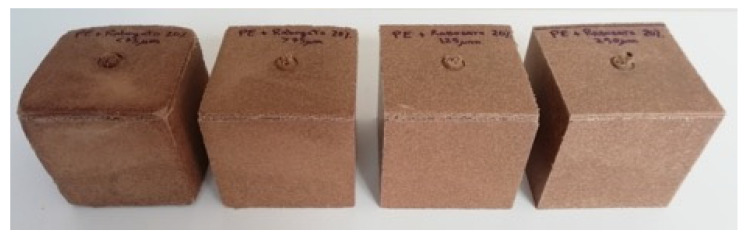
Rotomolded cubes with 20% weight of *Pennisetum setaceum* at different fiber sizes: D, C, B, and A (from left to right, respectively).

Composites with 20% of *Pennisetum* also showed some problems during molding, as observed in Figure 8, especially for those formulations with lower particles sizes.

#### 3.1.3. Composites Obtained with *Ricinus communis*

**Figure 9:**
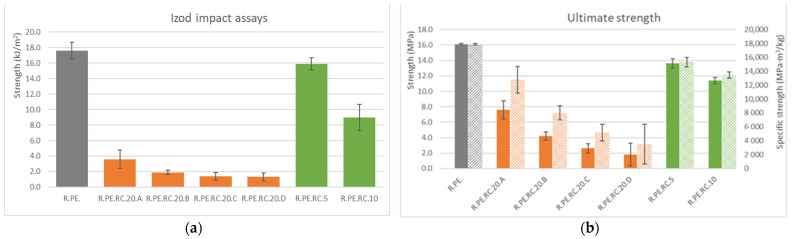

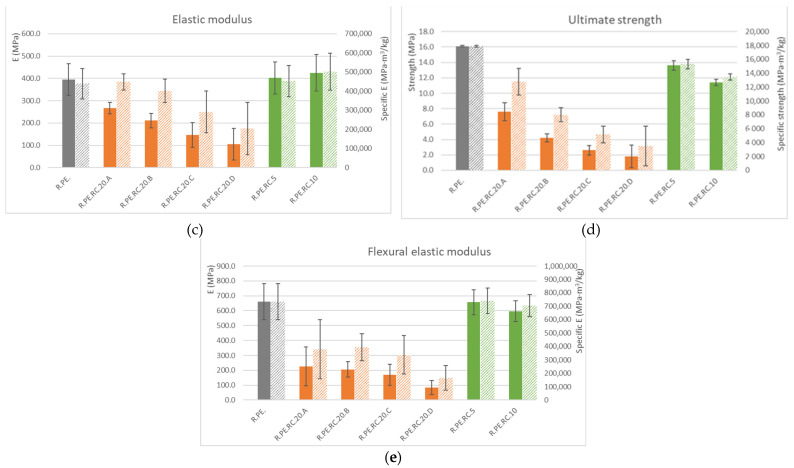
Summary of mechanical properties obtained for rotomolded *Ricinus communis* composites (solid bars show absolute properties, and lined bars show specific properties): (**a**) Izod impact assays, (**b**) tensile ultimate strength, (**c**) tensile elastic modulus, (**d**) flexural maximum strength, (**e**) flexural elastic modulus.

The trends already mentioned for *Arundo* composites were also observed for *Ricinus*, with a reduction in mechanical properties that was not as drastic as for *Pennisetum* ones. Figure 9 shows the mechanical properties obtained for *Ricinus* parts. Tensile specific elastic modulus was not modified due to the introduction of the *Ricinus* particles in sizes A and B, obtaining results similar to those shown by neat PE and the composites with non-sieved fibers and rates of up to 10%.

**Figure 10:**
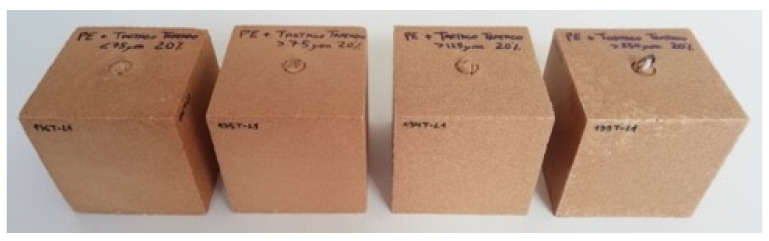
Rotomolded parts with 20% weight of *Ricinus communis* at different fiber sizes: D, C, B, and A (from **left** to **right**, respectively).

The levels of deviation from the mechanical tests were similar among series, which was in line with the aesthetics of the obtained parts (Figure 10); for these composites, the homogeneity was better than that of the other composites obtained in this research.

### 3.2. Composites with Treated Fibers

#### 3.2.1. Composites Obtained with *Arundo donax* L.

**Figure 11:**
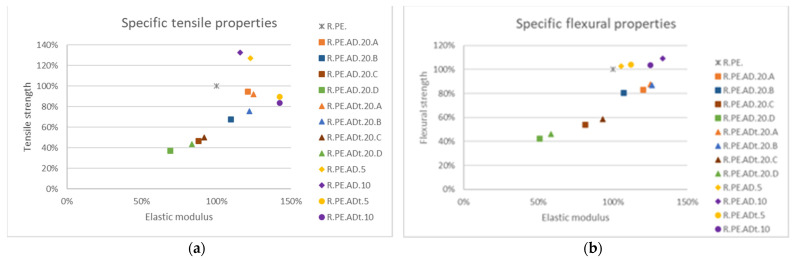
Variation of specific tensile (**a**) and flexural (**b**) properties for all obtained *Arundo* composites in reference to neat PE.

Figure 11 shows the changes in specific tensile and flexural properties, taking neat polyethylene samples as a 100% reference; it can be observed that only composites with smaller particles sizes (C and D) provided lower elastic moduli than PE for both tensile and flexural tests. It could also be observed that composites with treated fibers had higher elastic moduli in all cases. It appeared that composites with 5 and 10% of fiber provided the best mechanical properties, although sieved fibers at bigger sizes achieved improvements of both tensile and flexural elastic moduli without significant losses in tensile and flexural strength, allowing for their weight substitution in 20% polymer matrix by the natural fiber.

**Figure 12:**
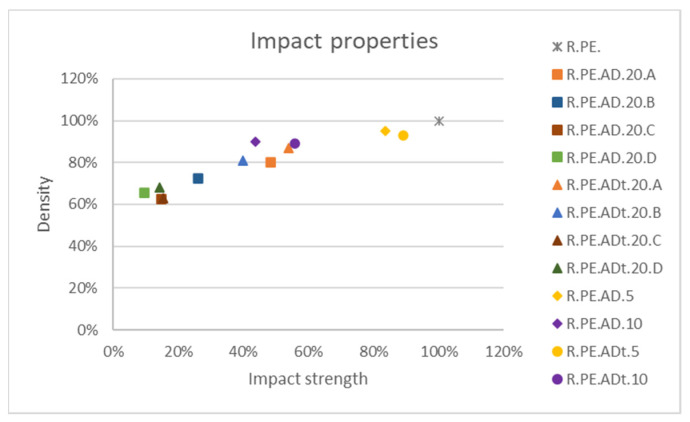
Impact strength variation for *Arundo* composites.

On the other hand, only composites with 5% of *Arundo* fibers showed acceptable impact strengths compared to neat PE. It was also observed that 10% and 20% composites containing A-fiber (sieved fibers at bigger sizes) had similar behavior (Figure 12). Thus, in those cases where impact resistance is not of key importance, up to 20% of fibers could be used without significantly modifying this property (vs. 10% composite).

#### 3.2.2. Composites Obtained with Pennisetum Setaceum

**Figure 13:**
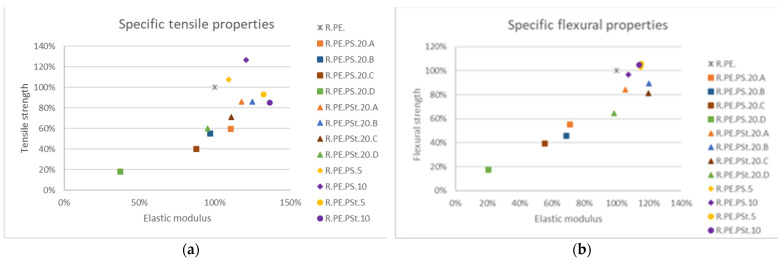
Variation of specific tensile (**a**) and flexural (**b**) properties for all obtained *Pennisetum* composites in reference to neat PE.

*Pennisetum* composites did not show clear differentiation in properties, especially in flexural properties, attributed to the fiber sizes (Figure 13). In any case, chemical treatment seemed to have a great influence on mechanical properties, as both the tensile and flexural tests of composites with treated fibers had much better results than for composites with untreated fibers. Again, 5 and 10% composites provided the best mechanical properties, although 20% composites with the A and B fiber sizes (treated fibers) also showed good behavior, with increases in tensile and flexural elastic modulus and a small reduction in tensile strength.

**Figure 14:**
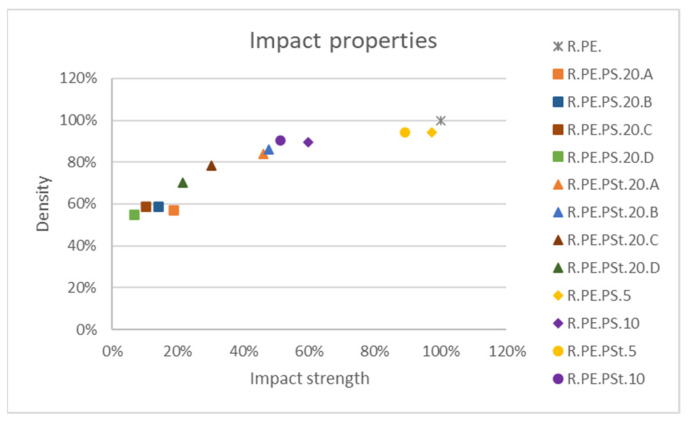
Impact strength variation for *Pennisetum* composites.

Similarly to observations made for the *Arundo* composites, only 5% *Pennisetum* samples allowed us to obtain parts without significant losses of impact strength (Figure 14). Composites with untreated fibers showed very poor impact behavior, as well as the lowest densities of this series of composites; fiber treatment led to the obtainment of denser composites, with the 20% composites (A and B sizes) having similar properties to the 10% composites, as also observed for reed composites.

#### 3.2.3. Composites Obtained with Ricinus Communis

**Figure 15:**
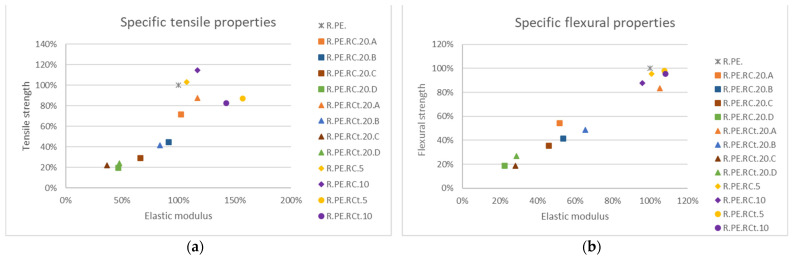
Variation of specific tensile (**a**) and flexural (**b**) properties for all obtained *Ricinus* composites in reference to neat PE.

Figure 15 summarizes the changes in mechanical properties for *Ricinus* composites. Tensile modulus was improved by over 50% with respect to neat PE when using treated *Ricinus*, while tensile strength was not modified (or slightly improved) when using untreated material (for 5 and 10% loadings). Only fibers classified as “A” and “B” provided similar elastic moduli to that of neat PE, though with reductions in tensile strength (only samples with treated fibers and the A size could be considered close to the resin). Similar observations could be made for flexural properties. Chemical treatment seemed to have less influence on this material than on the remaining fibers used in this research.

**Figure 16:**
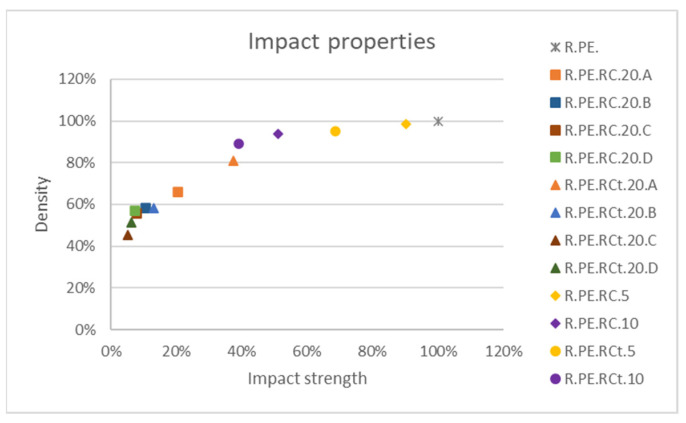
Impact strength variation for *Ricinus* composites.

Finally, impact assays (Figure 16) showed that only composites with 5% untreated *Ricinus* fibers could be considered to have good impact absorption, as all remaining composites for this series showed a drop of larger than 30% in this property. Fiber sieving did not seem to improve the impact behavior of these composites, although as already commented, longer fiber sizes provided the best values (among the low obtained values).

### 3.3. Comparison of Vegetal Species

**Figure 17:**
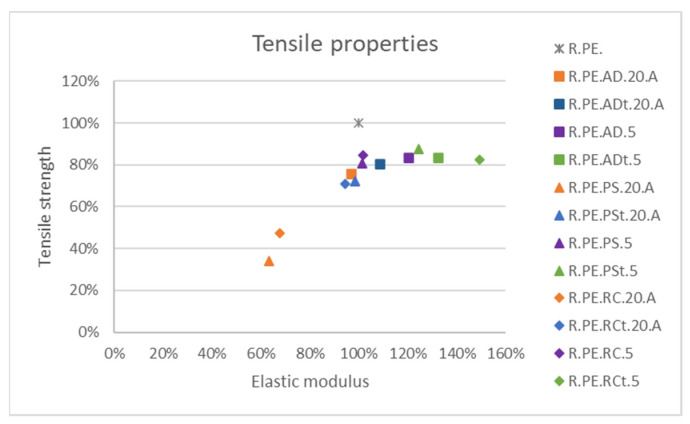
Comparison among tensile property variation for the different fibers.

This section serves as comparison of properties obtained for the three species at 5% and 20% loadings with longer fiber sizes (>250 μm; size A), which were the combinations found to have better mechanical properties. Figure 17 shows a comparison for tensile properties, where it can be observed that most of the formulations achieved around 80% of the tensile strength of neat PE, while only composites with 20% of untreated *Ricinus* and *Pennisetum* fibers show important decreases in modulus and strength. From this graph, it can be concluded that, although 5% treated fiber composites had the best tensile properties, composites can be produced with up to 20% of A size *Arundo* fibers while keeping similar tensile properties.

If specific properties are considered, only 20% *Ricinus* composites showed considerably lower elastic moduli, while only 20% *Pennisetum* provided significantly lower tensile strength. The remaining composites provided higher elastic moduli and lower tensile strength than those of PE (not reaching 20% of reduction).

**Figure 18:**
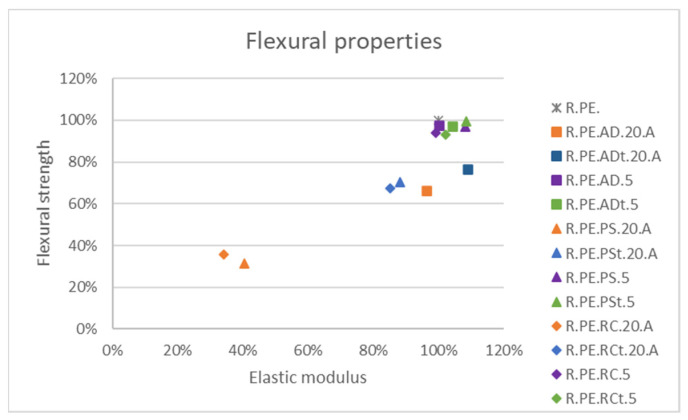
Comparison among flexural property variation for the different fibers.

Regarding specific flexural properties, *Ricinus* and *Pennisetum* at 20% again showed a drastic reduction in both strength and modulus, while the remaining combinations provided similar levels of these parameters. It is interesting to note that 20% of *Arundo* (treated or untreated) showed the best elastic modulus of all the materials tested, with a reduction of less than 20% in maximum strength, while the 5% composites provided similar levels to PE for both properties. A comparison of absolute flexural properties led to similar conclusions, although clearer differences among series could be observed (Figure 18); *Arundo* composites provided the best flexural properties at 20% fiber loading. The chemical treatment of fibers did not appear to have any influence on flexural properties for the 5% composites but allowed for the increase of very significantly properties for the 20% composites. For the 20% composites, only treated *Arundo* could be considered to have provided acceptable properties, as the modulus remained similar to the polyethylene modulus (even slightly higher) without a great reduction in maximum strength.

**Figure 19:**
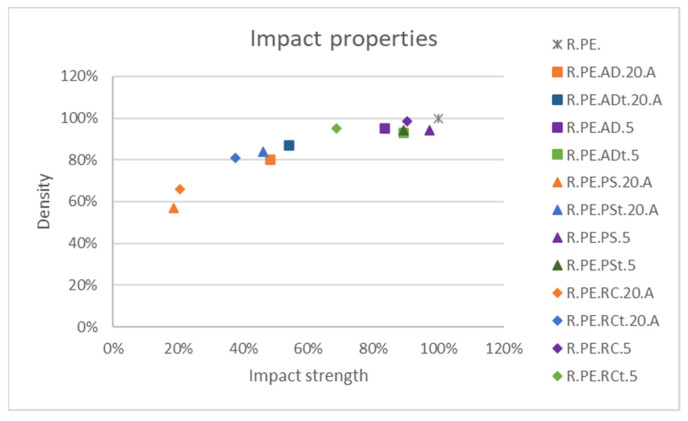
Comparison among impact property variation for the different fibers.

Finally, for impact properties (Figure 19), it was clear that only the 5% composites were able to get close to the values for PE; the untreated *Pennisetum* composites showed the best values. These were also the composites with the smallest decreases in density.

### 3.4. Morphology of Composites

**Figure 20:**
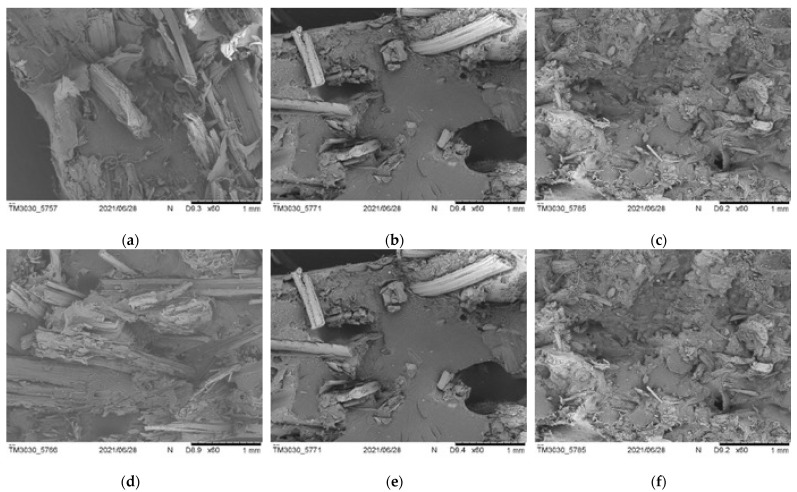
SEM images for 10% composite breakage sections ((**a**–**c**): untreated fibers; (**d**–**f**): treated fibers): *Arundo*, *Pennisetum*, and *Ricinus* ((**a**) and (**d**), (**b**) and (**e**), (**c**) and (**f**), respectively).

SEM pictures taken at ×60 and ×200 magnification allowed us to obtain clear views of the composites’ internal structures, presence of voids, and fiber/matrix adhesion. Composites with lower amounts of fibers—especially *Arundo* composites—showed compact structures, with low numbers of voids (Figure 20). This explains the better mechanical properties of the *Arundo* composites. NaOH-treated fibers showed similar structures to the untreated ones, although composites also showed lower amount of voids in general.

**Figure 21:**
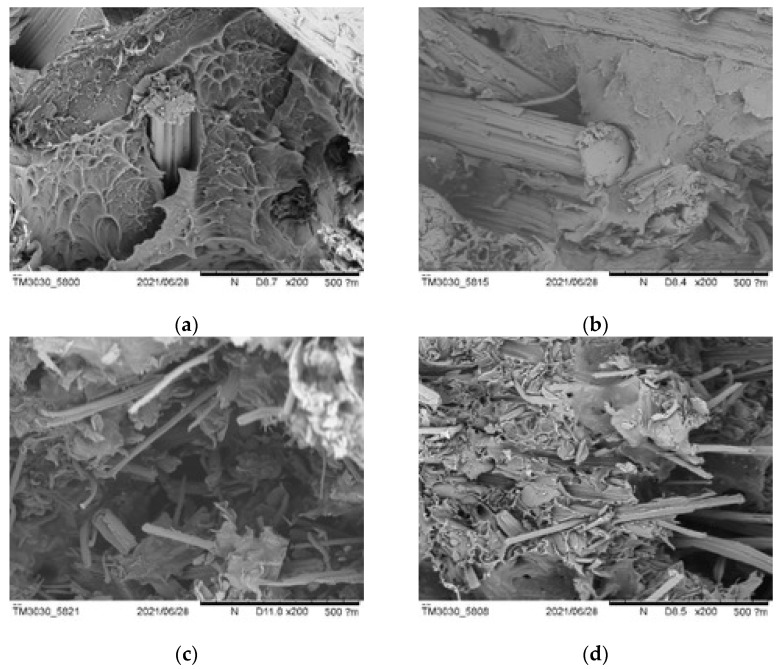
SEM images for 20% *Arundo* composite breakage sections: untreated and treated ((**a**) and (**c**), (**b**) and (**d**), respectively); A and D sizes ((**a**) and (**b**), (**c**) and (**d**), respectively).

The 20% composites, especially those with the smaller particle size (D), showed high percentages of voids for the three fibers; this explained the poor mechanical behavior of these composites. Figure 21 shows SEM pictures of the *Arundo donax* composites for the A and D fiber sizes (bigger and smaller, respectively). Pictures of the different composites are found in the Supplementary Material (Figures S1–S3).

A high number of voids and a lack of continuity in the composites with fibers below 75 microns, together with fiber clusters, could be observed. Pull-out of fibers could be observed for all fibers (both untreated and treated); the treatment of the fibers seemed to have a beneficial effect on fiber/matrix adhesion and/or fiber distribution, thus leading to better mechanical properties, mainly for the D-sized fibers.

## 4. Discussion

In general terms, it was observed that mechanical properties were affected by the introduction of the fibers within the PE matrix. As rotational molding consists of a low-shear process, it is not easy to obtain a homogeneous composite, and fiber agglomerations and segregations (which directly affect mechanical properties) were formed [31].

Density was greater for the 5 and 10% composites, with no significant differences versus the PE matrix. For the 20% composites, it was observed that composites with treated fibers showed higher values of density than the untreated ones, as also found by Hanana [32]. As observed in other studies, larger loadings of fibers led to parts with lower bulk density due to the greater porosity of the composites [28]. As already mentioned, composites with smaller fiber sizes showed higher number of voids and defects, which also explained their lower densities [33,34]. Untreated *Pennisetum* composites showed the lowest density values, with no significant differences, also explaining the poor mechanical results obtained for these materials. As observed in Figure S1 for *Arundo*, the composites with smaller sizes had a large number of voids; 10% composites showed almost no bubbles, while 20% composites with untreated fibers of size D had a large number of bubbles visible in the part surface and the cross section. The frequency of these defects was gradually reduced as the fiber size increased. Similar observations were made for remaining fibers (Figures S1–S3: S1 for *Arundo* composites, S2 for *Pennisetum*, and S3 for *Ricinus*).

As observed from the graphs included in the Results section, impact properties were greatly affected by the introduction of foreign materials. However, it was seen that the 5% composites had no reduced impact strength. Other authors have reported larger drops for polyethylene composites; for example, banana, and abaca fiber composites showed a decrease in impact energy close to 90% with a 5% of fiber [35], while others found similar behavior for 5% composites with bamboo and LLDPE [36] and reductions over 50% with respect to neat PE for sisal and cabuya composites (at a maximum loading of 7.5%) [23]. Cisneros–López et al. [31] were able to obtain a biocomposite with 10% of fibers with a slightly better impact behavior than an LMDPE matrix, and they observed important reductions with larger amounts of fibers when treated with a compatibilizer (impregnated with a maleic anhydride grafted polyethylene—MAPE). Some researchers have observed that composites with longer fibers provide better impact properties, as there is more distance to fiber pull-out and more energy is required [31]. The results shown in this manuscript confirmed this trend: the longer the fibers, the better the mechanical properties.

In this study, tensile elastic modulus was increased with the introduction of fibers, while maximum strength (both for tensile and flexural tests) was reduced. Fiber size was demonstrated to play an essential role in the mechanical properties of rotomolded composites; lower size fibers provided the poorest mechanical properties. Only fibers with a size of over 250 μm allowed us to obtain composites with good mechanical properties (flexural and tensile) at 20% loading. Hanana also found particles of 355–500 µm to prevent the formation of voids and bubbles, thus positively affecting mechanical properties [37]; specifically, composites with 30% of wood fiber increased elastic tensile modulus by up to 73% versus PE (for MAPE-impregnated fibers within this size range), improving impact properties by up to 50% and tensile strength by up to 114% when comparing 355–500 and 125–250 µm composites [32]. This range of particles sizes was similar to the most important fraction of the polymer for rotational molding, which allows for the improvement of the blending and limiting fiber segregation [38]. Thin particles (50 µm) avoid the coalescence of polymer grains, thus hindering the process of part formation [34], with this effect being more pronounced for larger amounts of filler (these authors did not manage to shape composites with over 15% of these thin particles). Elastic modulus is generally improved for composites with low fiber loadings; for example, composites with up to 10% of agave fibers were found to improve both flexural and tensile stiffness, while larger fiber loadings led to poor properties [31]. For some of the composites obtained in this research, these properties were comparable to those of neat PE; when comparing them in terms of density (specific properties), composites generally showed higher elastic moduli than PE.

Test parts made with three layers (with the fibers in the central layer with 5% of abaca fibers) showed a flexural modulus of around three times higher than PE samples while also increasing flexural maximum strength by almost 100% [35]. At this point, it should be noted that, even if mechanical properties are better than those obtained in this research, the manufacturing method is much more complex because the three layers should be added sequentially; on the other hand, the total content of fibers in these samples was 1.25%. Other authors obtained a flexural elastic modulus of around 600 MPa, which was two times higher than for neat PE, when using 10% of agave fibers [27] and 10% of abaca fibers [26], in single layer structures. These authors also applied an NaOH treatment to fibers, obtaining lower results than for untreated fibers due to formation of fiber clusters; these agglomerations were avoided in this research by sieving the fibers after the treatment, which allowed us to obtain more homogeneous composites while increasing the amount of fibers used. Wang produced composites with 10% of flax fibers, obtaining flexural and tensile properties quite similar to PE ones and only slightly increasing (10%) the tensile strength when treating the fibers with silane [25]. Torres made similar observations (unchanged tensile properties and very significant losses in impact energy absorption) with lower amounts of cabuya and sisal fibers [23]. The incorporation of grafted polyethylene with maleic anhydride was found to allow one to increase the tensile elastic modulus by 50% for 20% agave or coir composites (compared to a PE matrix) [29], while impact properties were not improved and decreased by around 30%. On the other hand, the pre-impregnation of fibers with maleated polymer (maleic anhydride grafted PE [31] or PLA [39]) has been proven to increase fiber distribution and enable the obtaining of composites with lower amounts of voids (thus with lesser porosity), with significant improvements in both tensile and flexural elastic modulus, as well as reductions in tensile and flexural strength, for up to 20% agave fibers—although these properties were worse than those of untreated fibers. This improvement has also been seen in impact properties [31]. MAPE was also used by Hanana [32], who demonstrated better adhesion between matrix and fiber. Sari and collaborators [40] also proposed the use of the plasma activation of PE to increase its adhesion with coir fibers, demonstrating improvements in impact strength—although only 5% fiber composites were produced. Finally, Oliveira et al. [41] were only able to introduce up to 5% of hemp fibers into a PE matrix without worsening the mechanical properties, even for NaOH- and MAPE-treated fibers.

It is generally accepted that high rates of fiber incorporation lead to agglomerations and poor homogeneity while increasing voids within the composite, which explains the worse mechanical properties for composites with more than 20% of fibers in rotomolding [31,36].

On the other hand, the composition of fibers is also known to have an effect on the final behavior of composites, with cellulose often seen as the component with the best mechanical properties. For the fibers used in this research, the largest cellulose content was found in *Arundo*; composites with this fiber were also the ones with the best mechanical properties (data not included in this paper). *Pennisetum* has the lowest cellulose content, and their composites also showed the poorest mechanical properties. However, the NaOH-treated fiber content in cellulose was greater than that of the untreated ones, mainly because of the hemicellulose removal, with similar values found for the three species (55.3 ± 0.8, 52.5 ± 0.9, and 50.6 ± 0.5% for *Arundo*, *Ricinus*, and *Pennisetum*, respectively). Differences in mechanical behavior were still observed, while treated fiber composites provided, in general terms, improved mechanical properties when compared to untreated ones. In summary, though composition is important for the final properties of a composite, it is not the only affecting factor; in this research, it was shown that fiber sizes seemed to have more influence than composition, as the porosity of the composite was also a factor with great weight.

## 5. Conclusions

Fiber sieving allows one to increase the amount of fiber included in a rotomolded composite, reaching values up to 20% without significant reductions of mechanical properties when compared to 5% loaded composites or even to neat PE.

NaOH-treated fibers generally provide better mechanical properties than untreated ones.

Impact properties were found to be greatly decreased as a consequence of fiber incorporation into the PE matrix, although composites with 5% fiber can be considered to provide acceptable impact strength.

Fiber size has been demonstrated to play an essential role in the mechanical properties of rotomolded composites: very fine fibers drastically reduce them, while fibers larger 250 μm enhance them, especially when high loadings of fiber are used.

## Figures and Tables

**Table 1:** Produced composites and corresponding codes.

Code	Description
R.PE.	Rotomolded PE (neat PE)
R.PE.AD.20.A	Rotomolded PE with 20 wt% *Arundo donax* fiber (P.S. >250 μm)
R.PE.AD.20.B	Rotomolded PE with 20 wt% *Arundo donax* fiber (P.S. 125–250 μm)
R.PE.AD.20.C	Rotomolded PE with 20 wt% *Arundo donax* fiber (P.S. 75–125 μm)
R.PE.AD.20.D	Rotomolded PE with 20 wt% *Arundo donax* fiber (P.S. <75 μm)
R.PE.AD.5	Rotomolded PE with 5 wt% *Arundo donax* fiber (non-sieved)
R.PE.AD.10	Rotomolded PE with 10 wt% *Arundo donax* fiber (non-sieved)
R.PE.PS.20.A	Rotomolded PE with 20 wt% *Pennisetum setaceum* fiber (P.S. >250 μm)
R.PE.PS.20.B	Rotomolded PE with 20 wt% *Pennisetum setaceum* fiber (P.S. 125–250 μm)
R.PE.PS.20.C	Rotomolded PE with 20 wt% *Pennisetum setaceum* fiber (P.S. 75–125 μm)
R.PE.PS.20.D	Rotomolded PE with 20 wt% *Pennisetum setaceum* fiber (P.S. <75 μm)
R.PE.PS.5	Rotomolded PE with 5 wt% *Pennisetum setaceum* fiber (non-sieved)
R.PE.PS.10	Rotomolded PE with 10 wt% *Pennisetum setaceum* fiber (non-sieved)
R.PE.RC.20.A	Rotomolded PE with 20 wt% *Ricinus communis* fiber (P.S. >250 μm)
R.PE.RC.20.B	Rotomolded PE with 20 wt% *Ricinus communis* fiber (P.S. 125–250 μm)
R.PE.RC.20.C	Rotomolded PE with 20 wt% *Ricinus communis* fiber (P.S. 75–125 μm)
R.PE.RC.20.D	Rotomolded PE with 20 wt% *Ricinus communis* fiber (P.S. <75 μm)
R.PE.RC.5	Rotomolded PE with 5 wt% *Ricinus communis* fiber (non-sieved)
R.PE.RC.10	Rotomolded PE with 10 wt% *Ricinus communis* fiber (non-sieved)

P.S. = particle size. An additional “t” in the code of the fiber, found in the manuscript or in results tables or graphs, refers to “treated fiber” (i.e., R.PE.ADt.10 refers to rotomolded samples with 10 % of *Arundo* treated fibers).

## Data Availability

The data presented in this study are available in the document, and in supplementary files (Table S1 and Figures S1–S3).

## Supplementary Materials

The following are available online at https://www.mdpi.com/article/10.3390/polym13132220/s1, Table S1: Mechanical properties of rotomolded composites (PE matrix and 20% fiber). A, B, C, and D refer to different fiber size distributions: A (>250 μm), B (125–250 μm), C (75–125 μm), and D (<75 μm), Figure S1: SEM pictures of *Arundo donax* composites, Figure S2: SEM pictures of *Pennisetum setaceum* composites, Figure S3: SEM pictures of *Ricinus communis* composites.
