# Supplementary material for: Valorization of Invasive Plants from Macaronesia as Filler Materials in the Production of Natural Fiber Composites by Rotational Molding
Source: Polymers (Basel). 2021 Jul 5;13(13):2220. doi: 10.3390/polym13132220 (PMC8272087; doi:10.3390/polym13132220)
Supplement: Supplementary file 1 [file polymers-13-02220-s001.zip › polymers-1228850-supplementary.pdf]

Supporting information:

Valorization of Invasive Plants from Macaronesia as Filler Materials in the Production of Natural Fiber Composites by Rotational Molding

Zaida Ortega [1,*], Francisco Romero [2], Rubén Paz [2], Luis Suárez [2], Antonio Nizardo Benítez [1] and María Dolores Marrero [2]

[1] Departamento de Ingeniería de Procesos, Universidad de Las Palmas de Gran Canaria, post code Las Palmas, Spain; antonionizardo.benitez@ulpgc.es
[2] Departamento de Ingeniería Mecánica, Universidad de Las Palmas de Gran Canaria, post code Las Palmas, Spain; francisco.romero@ulpgc.es (F.R.); ruben.paz@ulpgc.es (R.P.); luis.suarez@ulpgc.es (L.S.); mariadolores.marrero@ulpgc.es (M.D.M.)
* Correspondence: zaida.ortega@ulpgc.es

Table S1. Mechanical properties of rotomolded composites (PE matrix and 20 % fiber). A, B, C, D refers to different fiber sizes distribution: A (> 250 µm), B (125 – 250 µm), C (75 – 125 µm) and D (< 75 µm).

COMPOSITE	DENSITY	IMPACT		TENSILE (MPa)						FLEXURAL (MPa)					
	(g/cm³)	Strength (kJ/m²)		Ultimate strength		E		Yield strength		Ultimate strength		E_f		Yield strength	
R.PE.	**0,901**	**17,6**	± 1,1	**16,1**	± 0,1	**395,4**	± 71,2	**7,2**	± 1,3	**18,7**	± 2,1	**661,2**	± 120,7	**9,0**	± 1,7
R.PE.AD.20.A	**0,720**	**8,5**	±1,67	**12,2**	±0,16	**382,7**	±46,08	**3,7**	±0,72	**12,4**	±1,14	**637,1**	±72,05	**6,2**	±0,95
R.PE.AD.20.B	**0,654**	**4,6**	±0,69	**7,9**	±0,82	**314,9**	±30,09	**5,0**	±0,69	**10,9**	±1,08	**515,1**	±44,52	**6,3**	±0,75
R.PE.AD.20.C	**0,562**	**2,6**	±0,88	**4,7**	±0,80	**217,5**	±36,25	**2,1**	±0,75	**6,3**	±3,24	**336,1**	±143,15	**3,1**	±1,22
R.PE.AD.20.D	**0,589**	**1,7**	±0,64	**3,9**	±2,03	**179,0**	±89,12	**1,6**	±0,74	**5,2**	±4,14	**219,8**	±166,18	**2,8**	±2,26
R.PE.ADt.20.A	**0,785**	**9,5**	±1,39	**12,9**	0,37	**430,4**	±21,56	**5,3**	±1,64	**14,3**	±1,41	**721,8**	±78,66	**6,9**	±0,98
R.PE.ADt.20.B	**0,731**	**7,0**	±1,44	**9,9**	±0,74	**392,4**	±6,29	**5,7**	±0,64	**13,2**	±1,14	**677,8**	±127,12	**7,6**	±0,09
R.PE.ADt.20.C	**0,569**	**2,7**	±0,61	**5,1**	±1,16	**229,7**	±46,17	**3,4**	±0,72	**6,9**	±2,28	**389,3**	±103,67	**3,9**	±1,24
R.PE.ADt.20.D	**0,613**	**2,5**	±1,09	**4,8**	±1,92	**224,8**	±96,90	**2,6**	±1,07	**5,9**	±3,19	**263,6**	±143,59	**3,2**	±1,80
R.PE.PS.20.A	**0,515**	**3,3**	±0,57	**5,5**	±0,32	**249,5**	±31,85	**1,7**	±0,21	**5,9**	±0,68	**268,1**	±36,16	**3,4**	±0,64
R.PE.PS.20.B	**0,527**	**2,5**	±0,62	**5,2**	±0,46	**224,1**	±34,39	**2,7**	±0,91	**5,0**	±0,10	**266,4**	±13,29	**2,8**	±0,20
R.PE.PS.20.C	**0,527**	**1,8**	±0,36	**3,8**	±0,48	**202,7**	±36,86	**1,5**	±0,34	**4,3**	±1,84	**214,2**	±58,77	**2,5**	±1,06
R.PE.PS.20.D	**0,496**	**1,2**	±0,62	**1,6**	±1,35	**81,2**	±51,93	**0,5**	±0,33	**1,8**	±1,40	**74,1**	±64,55	**1,1**	±0,77
R.PE.PSt.20.A	**0,755**	**8,1**	±1,11	**11,6**	±0,41	**389,4**	±30,97	**4,5**	±1,31	**13,2**	±1,44	**583,4**	±42,37	**6,7**	±1,33
R.PE.PSt.20.B	**0,775**	**8,4**	±1,13	**11,9**	±0,58	**424,6**	±43,45	**6,1**	±1,03	**14,4**	±1,67	**681,4**	±95,69	**7,3**	±0,70
R.PE.PSt.20.C	**0,707**	**5,3**	±1,62	**9,0**	±0,75	**344,1**	±14,00	**4,1**	±0,92	**11,9**	±1,82	**621,3**	±103,76	**5,9**	±0,31
R.PE.PSt.20.D	**0,633**	**3,8**	±0,32	**6,8**	±0,57	**264,1**	±26,07	**4,0**	±1,26	**8,5**	±1,43	**457,0**	±16,70	**4,3**	±0,32
R.PE.RC.20.A	**0,595**	**3,6**	±1,19	**7,6**	±1,15	**266,9**	±25,29	**1,6**	±0,35	**6,7**	±3,88	**226,0**	±131,17	**3,5**	±2,38
R.PE.RC.20.B	**0,524**	**1,9**	±0,28	**4,2**	±0,54	**210,6**	±32,46	**1,5**	±0,28	**4,5**	±1,04	**206,0**	±52,22	**2,2**	±0,26
R.PE.RC.20.C	**0,503**	**1,4**	±0,48	**2,6**	±0,61	**146,5**	±55,23	**1,1**	±0,25	**3,7**	±1,95	**169,9**	±71,89	**1,7**	±0,71
R.PE.RC.20.D	**0,514**	**1,3**	±0,5	**1,8**	±1,48	**105,3**	±70,40	**0,8**	±0,48	**2,0**	±0,99	**84,8**	±47,22	**0,9**	±0,66
R.PE.RCt.20.A	**0,729**	**6,6**	±0,69	**11,4**	±0,33	**373,8**	±28,84	**3,6**	±1,31	**12,6**	±1,91	**563,1**	±105,99	**6,0**	±1,37
R.PE.RCt.20.B	**0,526**	**2,3**	±0,58	**3,9**	±0,48	**192,5**	±28,63	**2,2**	±0,61	**5,3**	±1,91	**252,6**	±72,35	**2,3**	±0,31
R.PE.RCt.20.C	**0,408**	**0,9**	±0,35	**1,6**	±0,81	**65,5**	±26,93	**0,6**	±0,18	**1,6**	±0,71	**84,4**	±49,88	**1,0**	±0,55
R.PE.RCt.20.D	**0,462**	**1,1**	±0,55	**2,0**	±1,46	**96,2**	±65,40	**0,7**	±0,40	**2,6**	±1,79	**97,8**	±64,60	**1,3**	±0,91

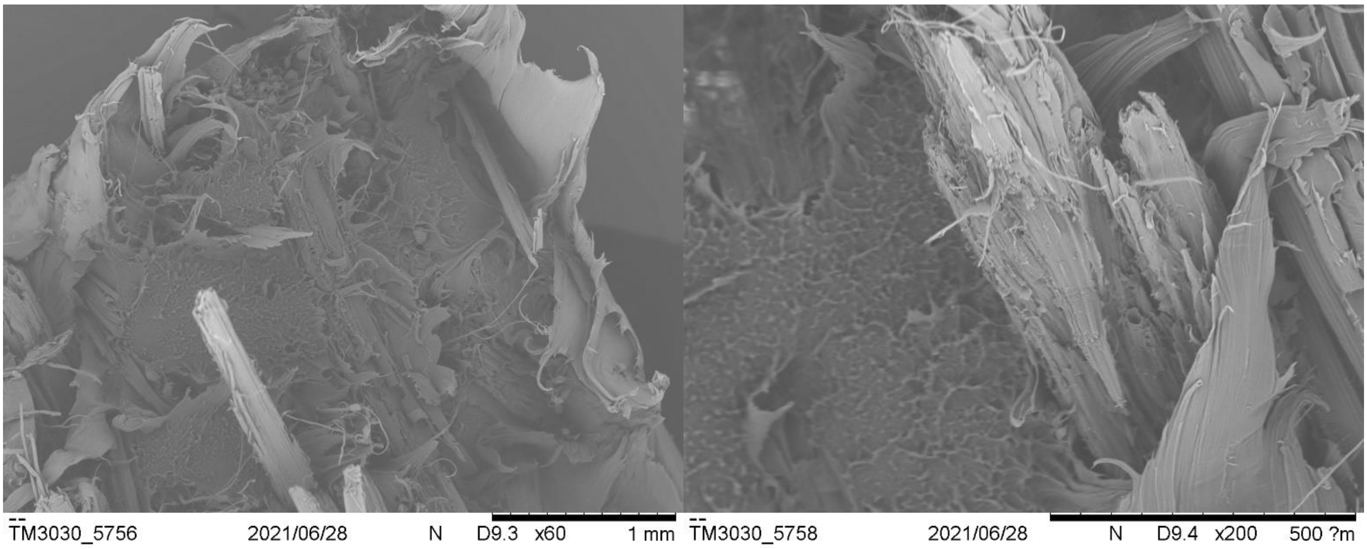
TM3030_5756 2021/06/28 N D9.3 x60 1 mm
TM3030_5758 2021/06/28 N D9.4 x200 500 ?m

(a)

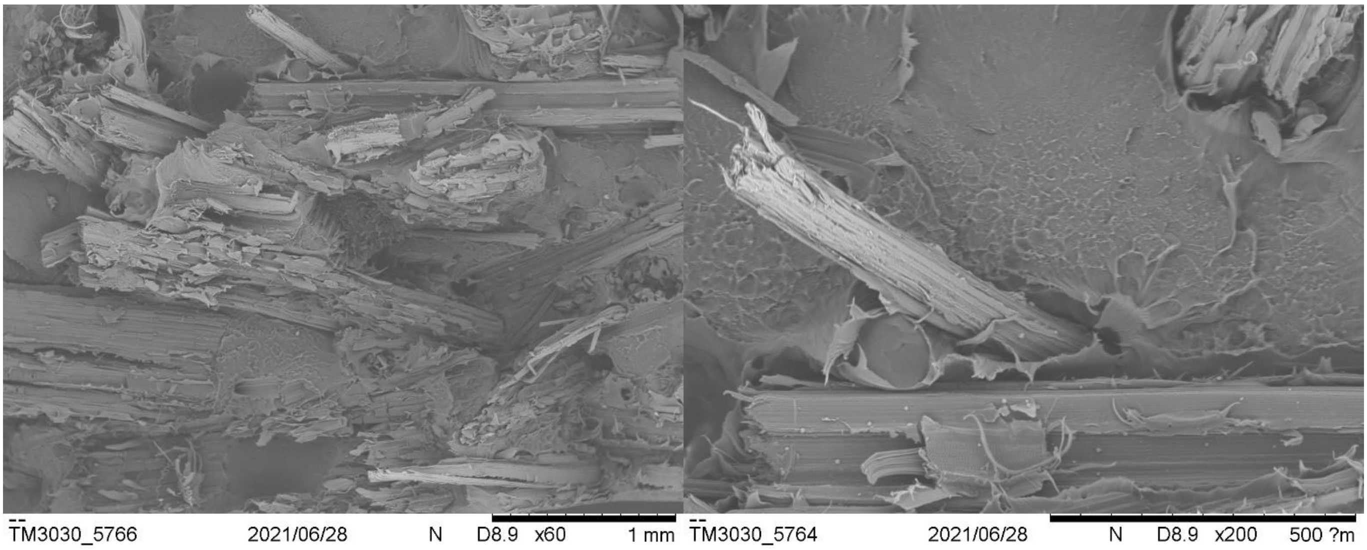
TM3030_5766 2021/06/28 N D8.9 x60 1 mm
TM3030_5764 2021/06/28 N D8.9 x200 500 ?m

(b)

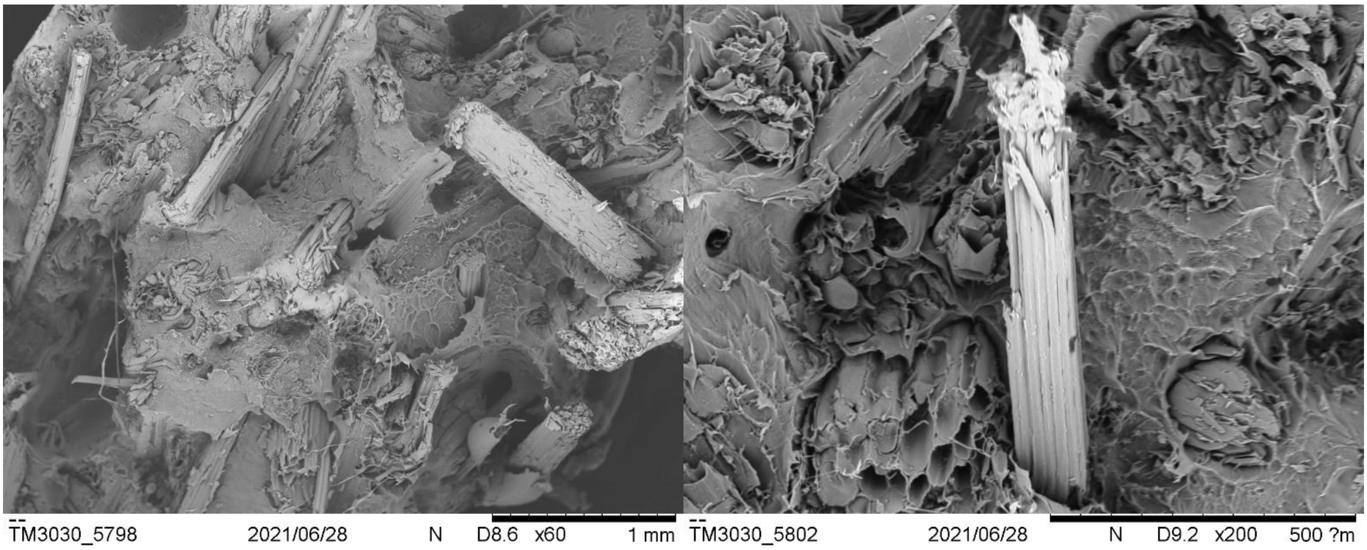
TM3030_5798 2021/06/28 N D8.6 x60 1 mm
TM3030_5802 2021/06/28 N D9.2 x200 500 ?m

(c)

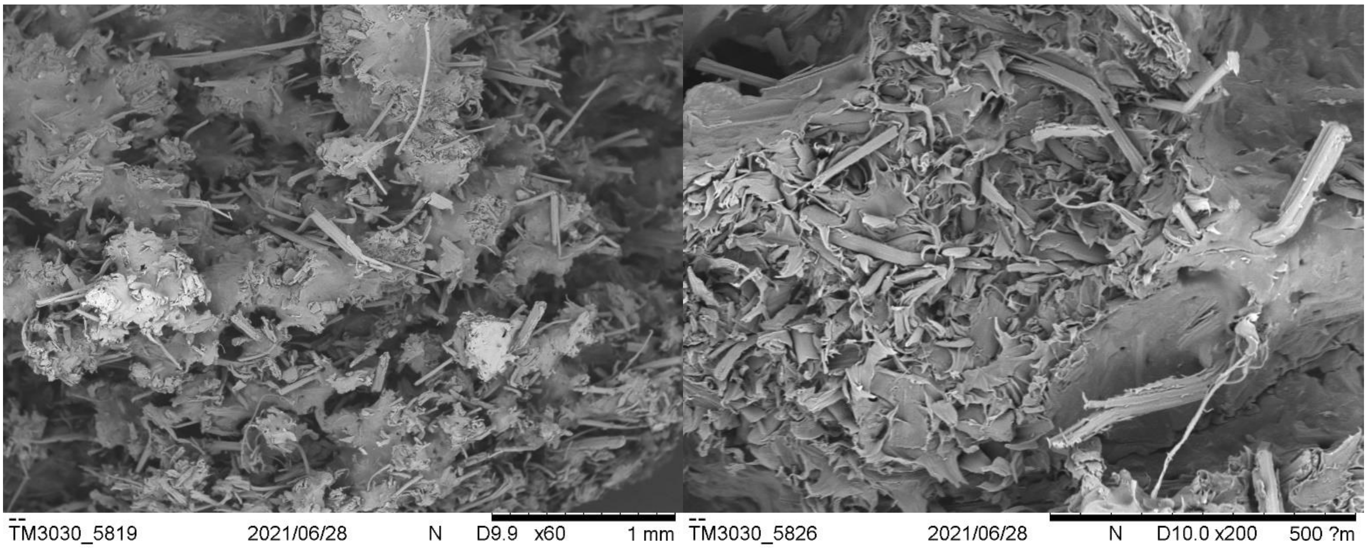

(d)

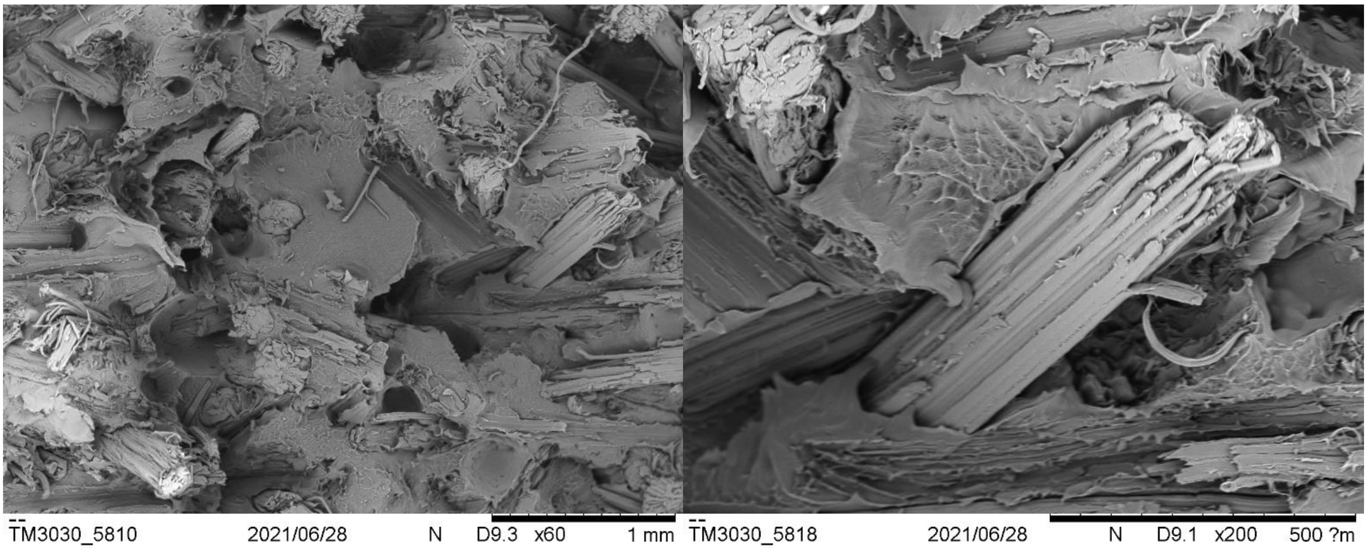

(e)

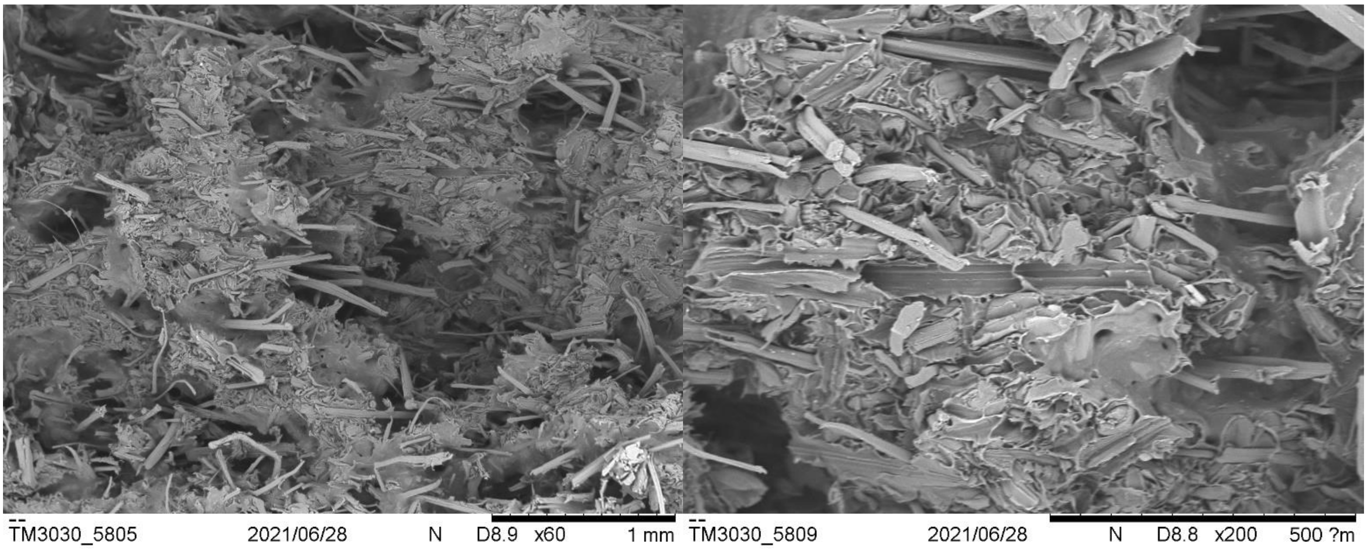

(f)

Figure S1. SEM pictures of *Arundo donax* rotomolded composites: (**a**) 10 % unsieved fiber, (**b**) 10% unsieved treated fiber, (**c**) 20% A-fiber (> 250 μm), (**d**) 20% D-fiber (< 75 μm), (**e**) 20% A-treated fiber (> 250 μm), (**f**) 20% D-treated fiber (< 75 μm)

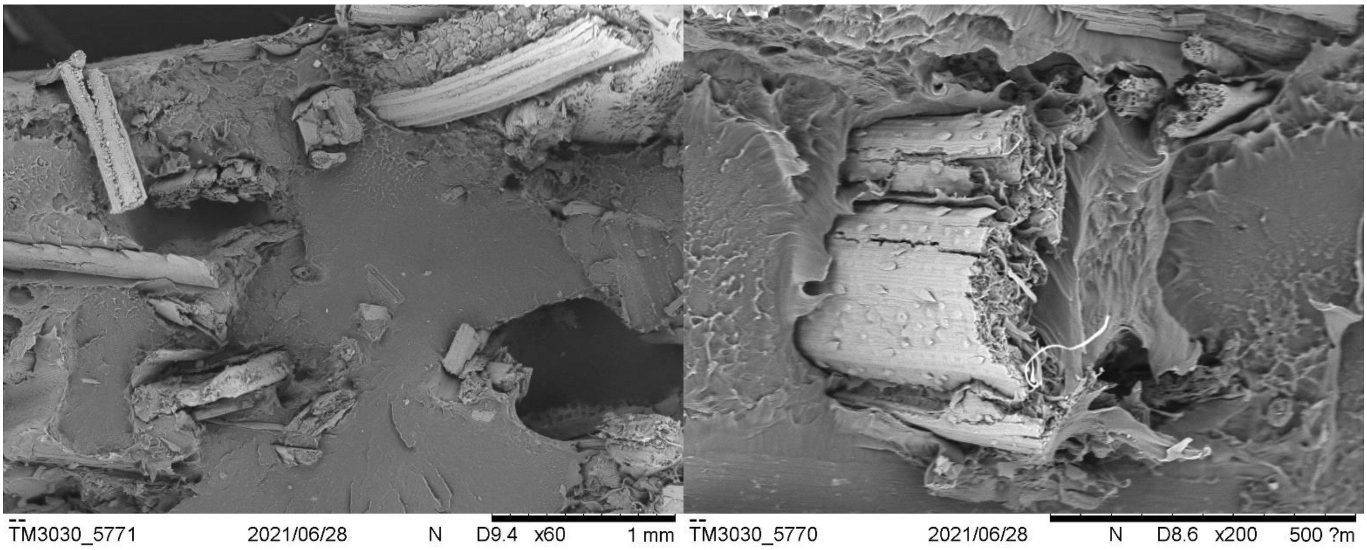
TM3030_5771 2021/06/28 N D9.4 x60 1 mm
TM3030_5770 2021/06/28 N D8.6 x200 500 ?m

(a)

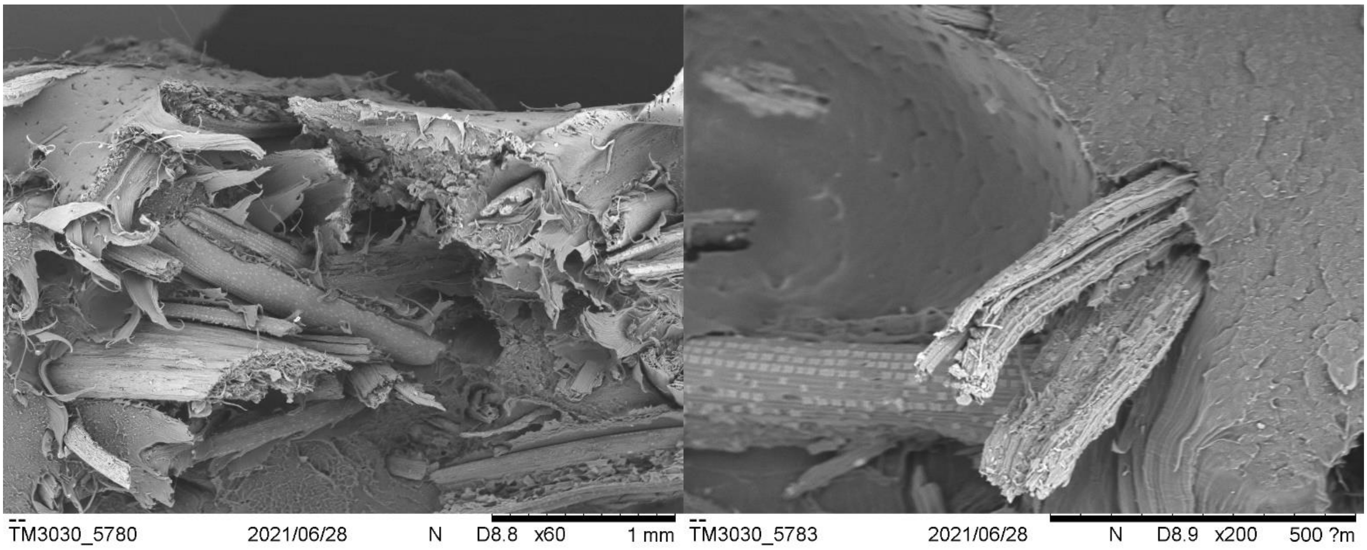
TM3030_5780 2021/06/28 N D8.8 x60 1 mm
TM3030_5783 2021/06/28 N D8.9 x200 500 ?m

(b)

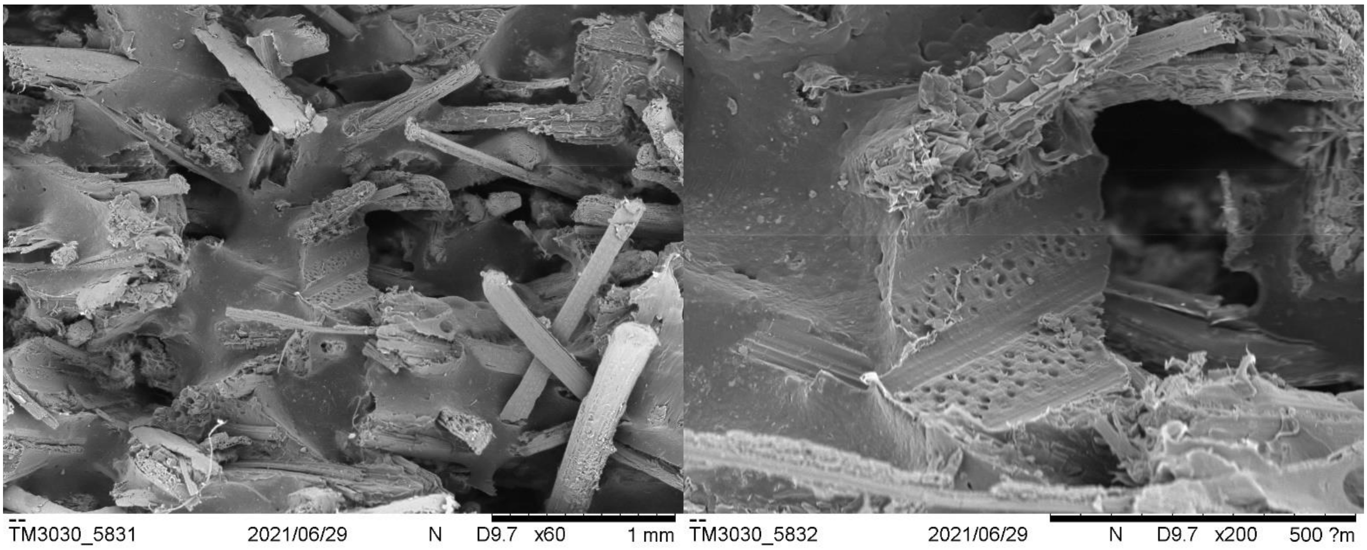
TM3030_5831 2021/06/29 N D9.7 x60 1 mm
TM3030_5832 2021/06/29 N D9.7 x200 500 ?m

(c)

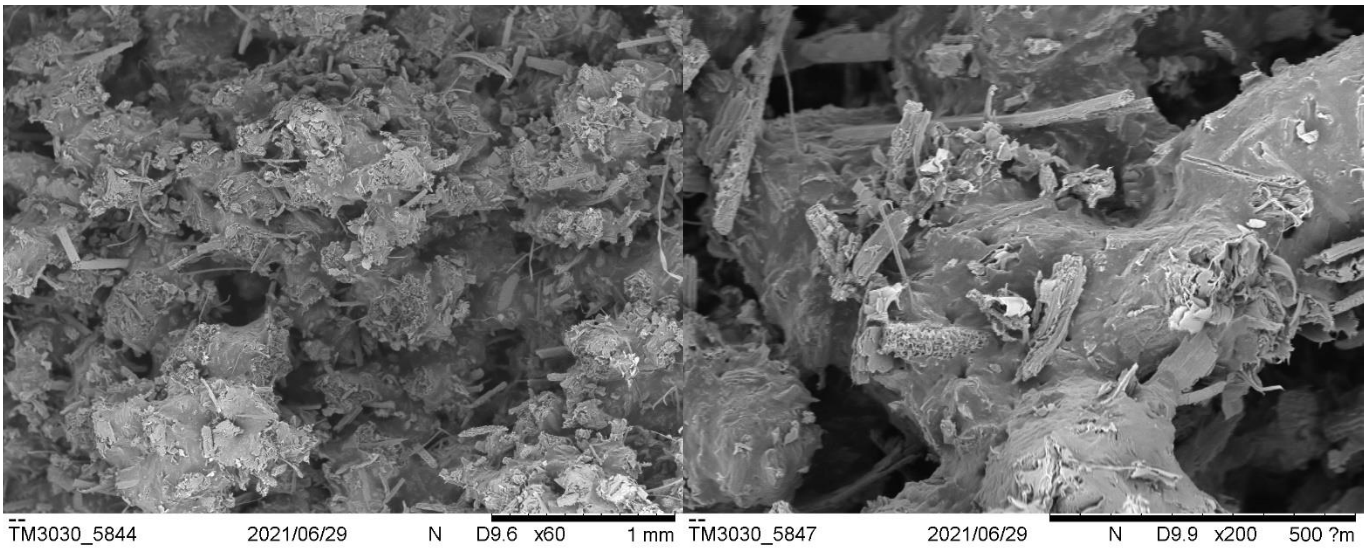

(d)

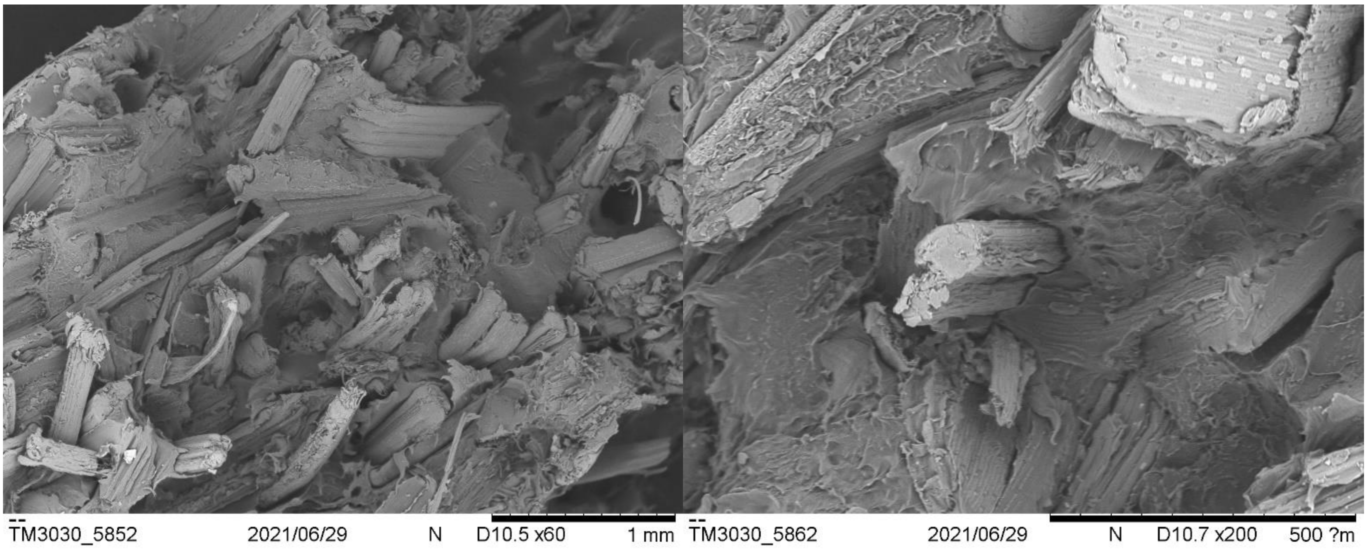

(e)

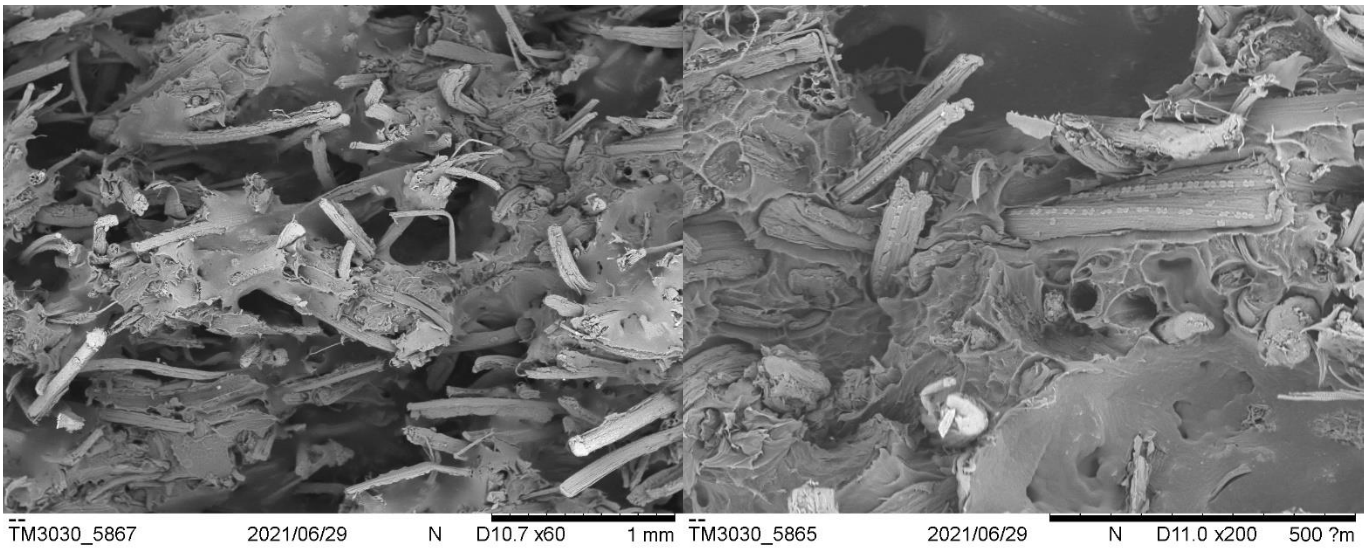

(f)

Figure S2. SEM pictures of *Pennisetum setaceum* rotomolded composites: (**a**) 10 % unsieved fiber, (**b**) 10% unsieved treated fiber, (**c**) 20% A-fiber (> 250 μm), (**d**) 20% D-fiber (< 75 μm), (**e**) 20% A-treated fiber (> 250 μm), (**f**) 20% D-treated fiber (< 75 μm)

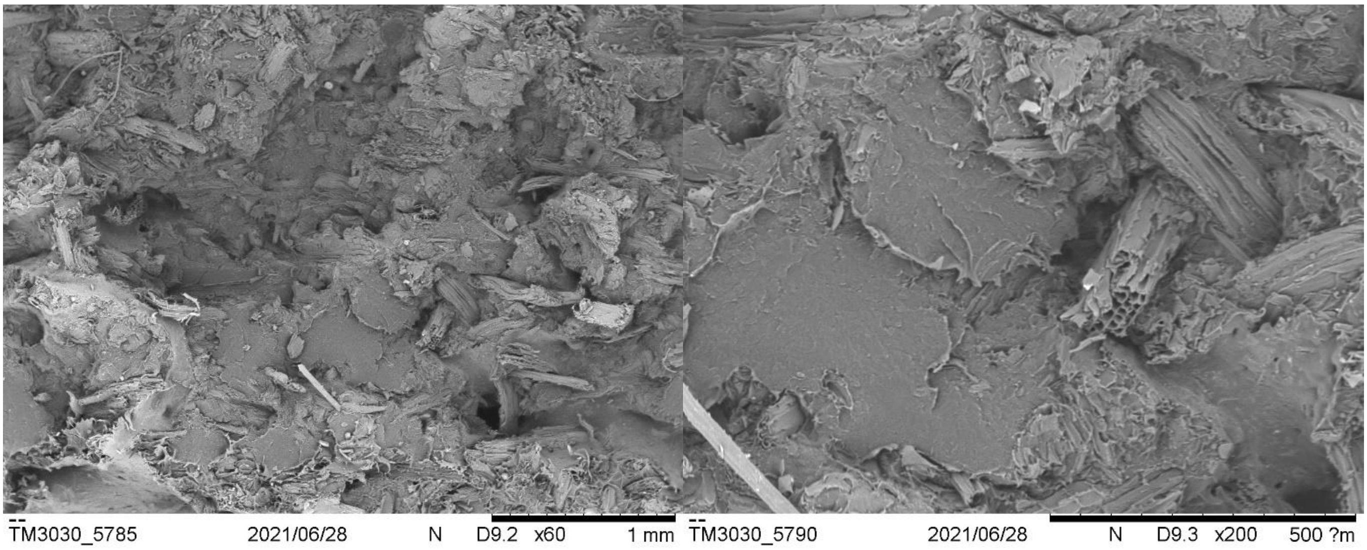
TM3030_5785 2021/06/28 N D9.2 x60 1 mm
TM3030_5790 2021/06/28 N D9.3 x200 500 ?m

(a)

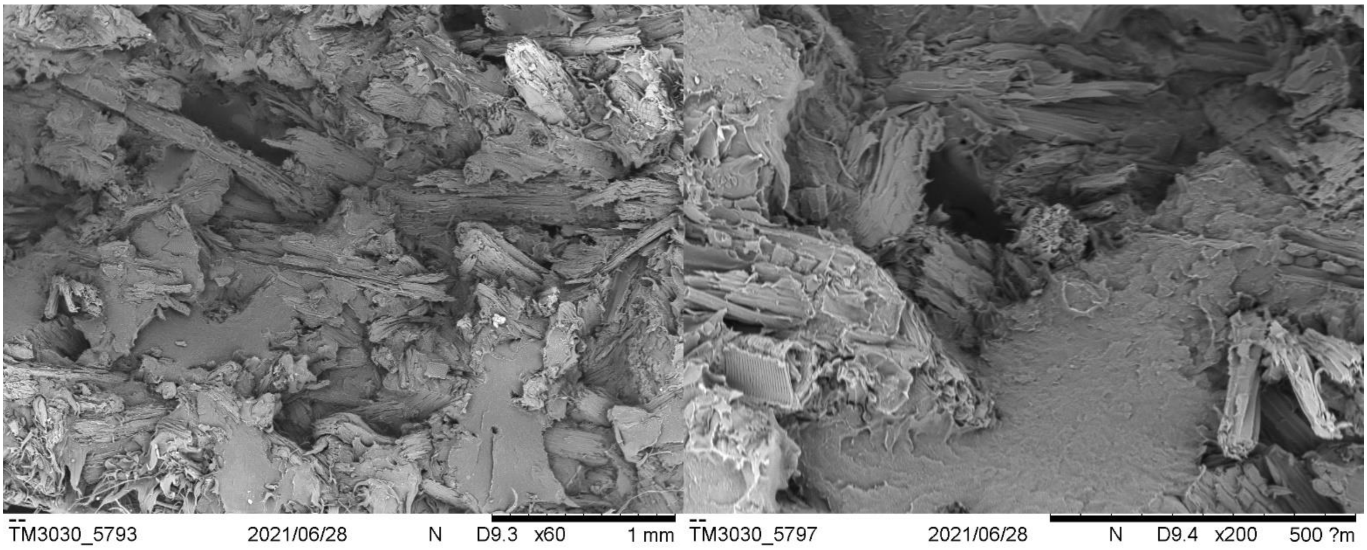
TM3030_5793 2021/06/28 N D9.3 x60 1 mm
TM3030_5797 2021/06/28 N D9.4 x200 500 ?m

(b)

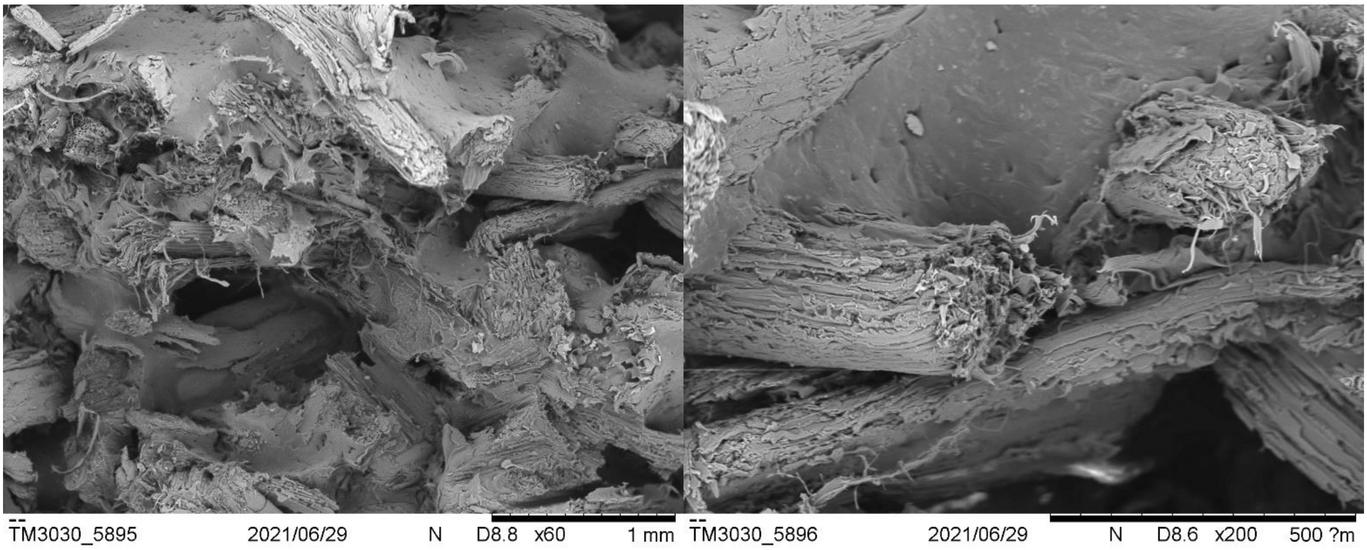
TM3030_5895 2021/06/29 N D8.8 x60 1 mm
TM3030_5896 2021/06/29 N D8.6 x200 500 ?m

(c)

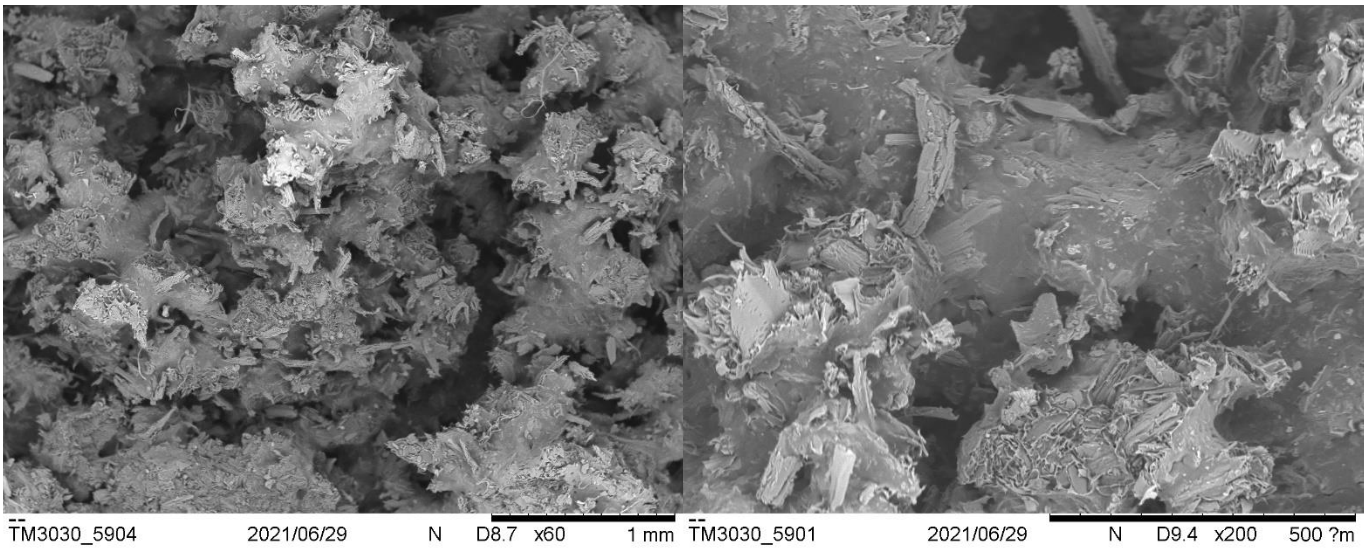

(d)

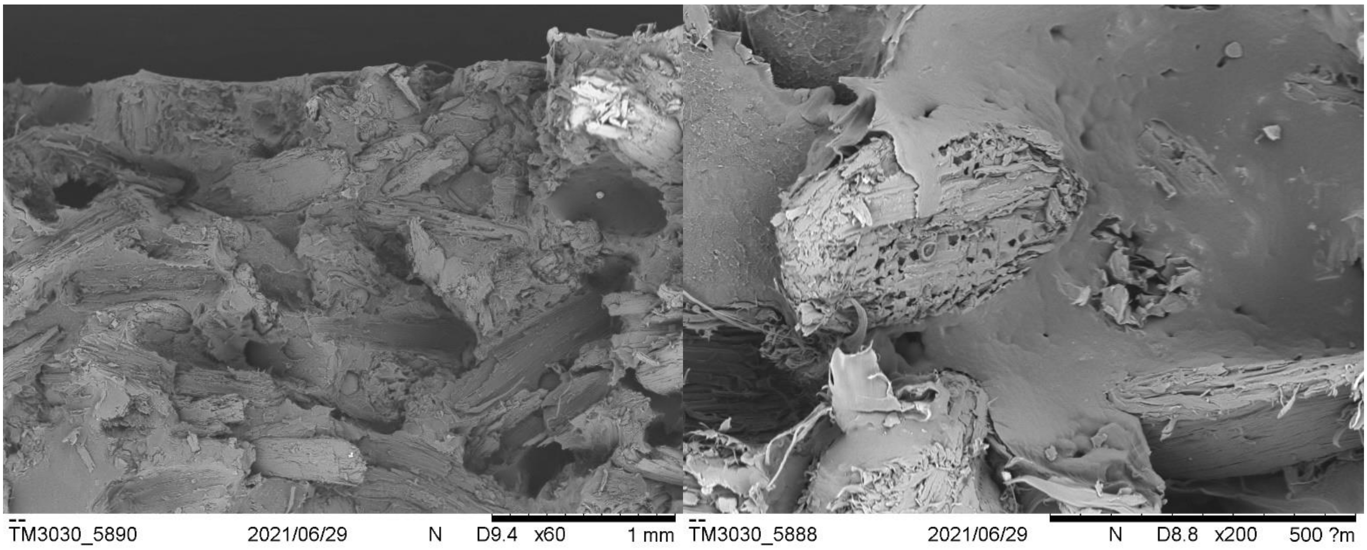

(e)

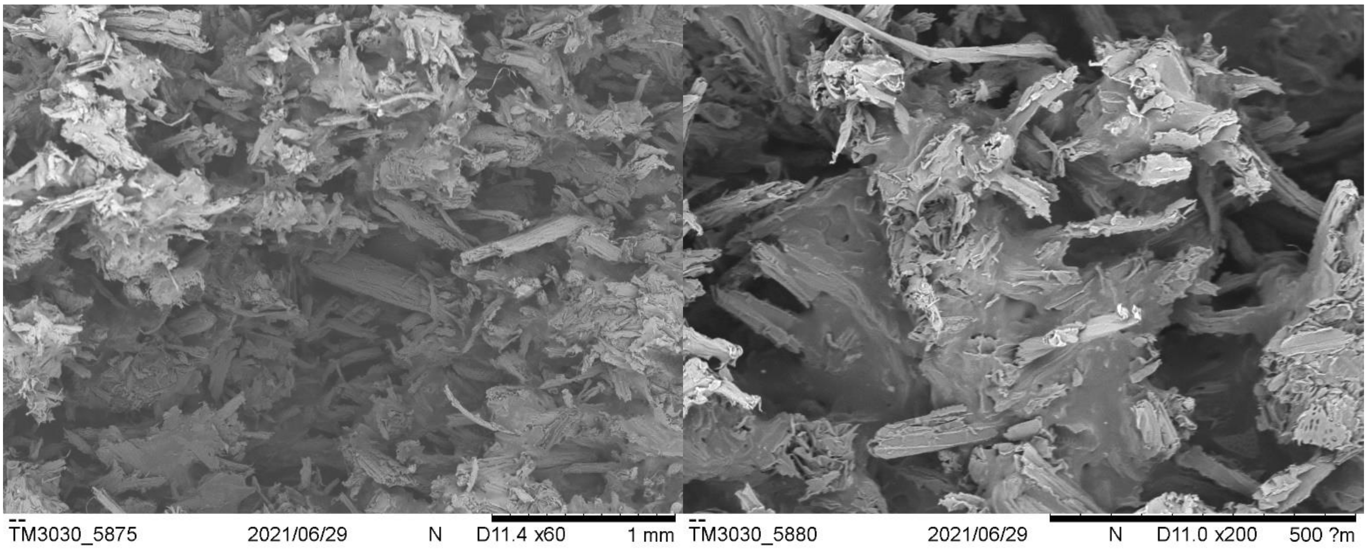

(f)

Figure S3. SEM pictures of *Ricinus communis* rotomolded composites: (**a**) 10 % unsieved fiber, (**b**) 10% unsieved treated fiber, (**c**) 20% A-fiber (> 250 μm), (**d**) 20% D-fiber (< 75 μm), (**e**) 20% A-treated fiber (> 250 μm), (**f**) 20% D-treated fiber (< 75 μm)
